# Intrinsic electrocatalytic activity of platinum grain boundaries: correcting measurement artefacts in scanning electrochemical cell microscopy (SECCM)

**DOI:** 10.1039/d5sc03829d

**Published:** 2025-09-10

**Authors:** Harry B. Swan, Lachlan F. Gaudin, Alannah J. Hunt, Cameron L. Bentley

**Affiliations:** a School of Chemistry, Monash University Clayton 3800 Victoria Australia cameron.bentley@monash.edu

## Abstract

Detailed understanding of the behaviour of an electrocatalyst is not only founded on the bulk properties of a given material, but must also consider contributions from surface structural motifs that often exist on the microscale or smaller. Often, surface features in the form of defects, including atomic step edges or grain boundaries, are considered to be important contributors to the total response of an electrocatalyst when performing a given process, though these are difficult to electrochemically characterise directly due to their physical size. Microscopic electrochemical techniques such as scanning electrochemical cell microscopy (SECCM) offer a convenient means of directly isolating and measuring these types of features, removing ambiguity about their properties and how they contribute to overall electrocatalyst behaviour. In this work, the intrinsic activity of grain boundaries on a polycrystalline platinum surface is investigated for the acidic hydrogen evolution reaction (HER) using SECCM. Through analysing the behaviour of 20 unique grain boundaries, it is established that surface tension effects can have a large conflating effect on apparent activity measured with droplet-based techniques such as SECCM, often producing false positive results in the search for electrochemically active sites. It is only through stringent surface preparation and surface area correction techniques that these features can be meaningfully analysed, with some suggested means of doing so demonstrated. Utilising these procedures, no grain boundaries with activities greatly exceeding that of the surrounding grains were able to be identified, even when working on the sub-micron length scale, suggesting that these sites, if they exist, are rare and would be unlikely to make a significant contribution to the macroscopic HER activity of platinum. Overall, this study presents some common misinterpretations that can result from the SECCM characterisation of grain boundaries and similar features with nanoscale probes, and establishes methods that could be taken in future works to ensure data is truly representative of their intrinsic properties.

## Abstract 2

Whilst surface defects such as grain boundaries on polycrystalline electrocatalysts may yield unique behaviour, their isolated electrochemical characterisation is complicated by abnormal surface–electrolyte wetting, requiring extra analytical rigour.

## Graphical abstract

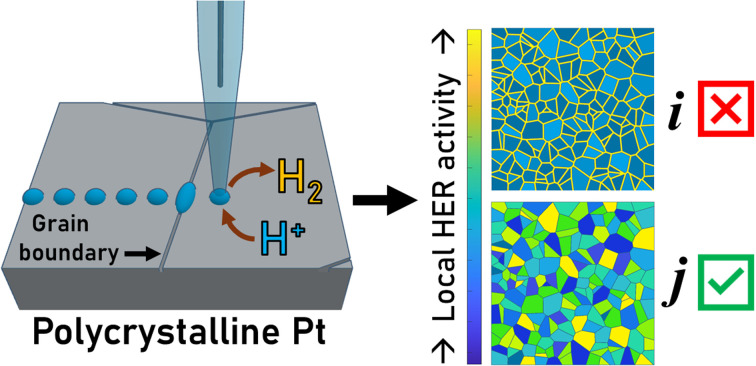

## Introduction

Modern electrocatalysis is largely concerned with the design, advancement, and use of electrochemically active materials. Much of this research and development relies upon building fundamental understanding of the relationship between electrocatalyst surface structure and electron transfer (catalytic) behaviour.^1–3^ With knowledge of the surface structures and features that give rise to high activity, stability and/or selectivity, more efficient electrode materials can be developed for implementation on an industrial scale. These principles also apply beyond the area of electrocatalysis, and are similarly important in designing electrode materials for other modern applications of electrochemical science, for example in battery cells.^4,5^

When studying electrocatalysts with a well-defined crystalline structure, a common theme is the determination of a structure–activity relationship of the electrode surface components. Often, this will refer to crystallographic (grain) orientation for polycrystalline metals,^6^ features such as step edges and basal planes in electrodes based on layered inorganic compounds,^7^ and shape and/or size when referring to nanoparticulate catalysts.^8^ In materials where the surface consists of regions of different crystallographic orientation (*e.g.*, grains in a polycrystalline metal surface), efforts to demystify variation in local electrochemical behaviour have often made substantial claims on the activity of grain boundaries.^9–12^ Frequently, odd or unexpected results in electrocatalyst tests showing increased or abnormally high activity have been attributed to the presence of grain boundaries on the analysed surfaces, usually with little to no direct electrochemical characterisation of the grain boundaries themselves. In the absence of other apparent reasons for inflated activity, it has often been assumed that the varying geometry and/or density of grain boundaries on a given macroscopic surface directly leads to the enhancement of overall electron transfer rate (electrocatalytic activity).

In many cases, the assumption that high activity or odd electrochemical behaviour can be attributed to the presence or density of grain boundaries is not unreasonable to make. A great deal of knowledge has been established regarding their importance in rates of corrosion, for example.^13,14^ Additionally, grain boundaries are fundamentally high-energy surface defects with unique and complex geometry—all traits that can easily influence the efficacy of electron transfer for inner-sphere or electrocatalytic processes.^15–17^ Despite this, the frequency of claims made for their increased electrocatalytic activity, and their relative lack of direct study make them interesting and important targets for thorough analysis.

A large part of the reason for speculative and not fully substantiated claims regarding grain boundaries comes from the fact that they are difficult to study *via* conventional macroscopic electrochemical methods. Preparing an electrode with a single isolated grain boundary (or multiple, well-defined boundaries) large enough to yield macroscopic effects presents significant difficulties,^18,19^ though this approach has been used in other fields to study other material properties.^20^ Instead, the abundance of microscale grain boundaries present in polycrystalline electrocatalysts can be conveniently studied by the use of electrochemical microscopy techniques, allowing for the direct and unambiguous characterisation of small surface features.^21,22^ Of particular relevance to this study is the technique known as scanning electrochemical cell microscopy (SECCM), which is a probe-based droplet technique capable of encapsulating small areas of an electrocatalyst surface (often smaller than 1 μm^2^) for entirely isolated electroanalytical study.^23^ This is ideal for the identification of nanoscale active sites and comparative analysis of catalyst surface regions.^24,25^ With the assumption that grain boundary activity, if meaningfully present, is accentuated when working on these scales, this technique is ideally suited for the unambiguous study of individual grain boundary behaviour.

In this work, initial studies of hydrogen evolution reaction (HER) behaviour on grain boundaries of polycrystalline platinum under acidic conditions were performed *via* SECCM. Using a polycrystalline platinum surface that had been prepared using standard protocols (*e.g.*, polishing and flame annealing), the SECCM scan procedure exhibited conflating effects that commonly yielded false positives in the search for ‘HER-active’ boundaries. These effects, caused by local surface area and droplet surface tension along grain boundary lines, artificially increased the current response on these boundaries. Demonstrated herein are procedures for the rigorous control of surface preparation and data correction techniques for avoiding these conflating errors. This study serves to present the origins of many pitfalls that may be encountered when studying polycrystalline material grain boundaries *via* droplet-based techniques such as SECCM, and suggests means of overcoming these to enable reliable analyses. It is possible that the conflating effects of surface area and surface tension have affected past and present studies of this nature, so these procedures (or similar) are suggested to be important inclusions in future work on the electrochemical study of grain boundaries.

## Results and discussion

### SECCM for the study of grain boundary activity

**Fig. 1:**
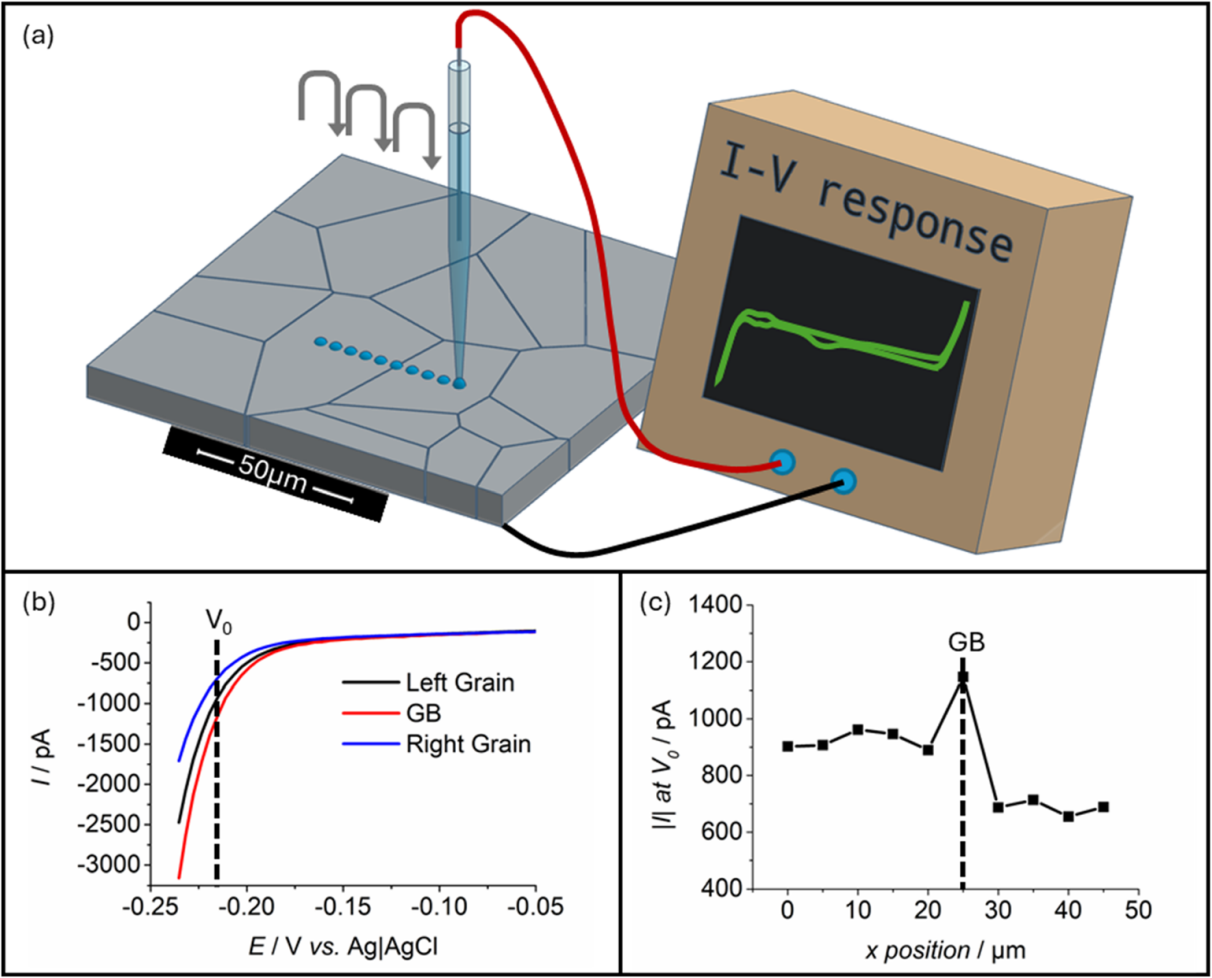
(a) Depiction of the basic layout of a typical hopping-mode SECCM setup, including the studied polycrystalline platinum surface, the nanopipette probe (including an inserted Ag|AgCl QRCE), and appropriate connections to a potentiostat. Also shown schematically are the controlled motion of the probe that produces a series of landings, and a schematic of a typical *I*–*V* response curve when cycling the platinum working electrode through its potential window in 0.5 M H_2_SO_4_. (b) Averaged LSVs experimentally obtained from hopping-mode SECCM measurements, found by sweeping cathodically at a scan rate of 4 V s^−1^ into potentials leading to the HER. A comparison is provided between the grain boundary (GB) response and the averages of the surrounding two grains. (c) A direct comparison of HER response currents at an arbitrary potential in the HER region (*V*_0_, −0.215 V *vs.* Ag|AgCl in this case), comparing responses on a linear landing-by-landing basis. The larger magnitude of current on the grain boundary itself, when compared to the surrounding areas, is what may typically be expected if an ‘active’ grain boundary is found. All experimental data were obtained using a probe with a tip diameter equal to approximately 1000 nm.

Herein, SECCM was utilised for the study of grain boundary electrochemical behaviour. Details on the experimental set up are provided in the Experimental section but are described briefly here to aid in understanding. As depicted in Fig. 1a, the configuration used for SECCM in this study employs a borosilicate glass nanopipette (of approximately 200 nm diameter) filled with 0.5 M H_2_SO_4_ electrolyte. This nanopipette is equipped with either a quasi-reference counter electrode (QRCE) or true reference electrode in what essentially constructs half of a two-electrode cell. To form a full electrochemical cell, the probe is advanced towards the substrate of interest until the droplet of electrolyte that naturally protrudes from the tip contacts the substrate surface. With full meniscus contact, the wetted area of the substrate can be used as the working electrode in voltammetric analysis of the system. With fine control of the position of the nanopipette, this analysis can be repeated by lifting the probe and landing on a different point of the substrate. Repeating this procedure in a ‘grid’ of landings forms the basis of what is known as ‘hopping-mode SECCM’, in which hundreds of voltammetric measurements can be taken on isolated regions (each smaller than 1 μm^2^) of the substrate surface at high resolution.^26^

The high-resolution capabilities and the complete isolation of individual measurements inherent to hopping-mode SECCM make it an ideal technique for the analysis of grain boundaries. In this study, the HER was analysed in this manner on a polycrystalline platinum substrate. This substrate was chosen due to platinum's ubiquity as an electrocatalyst and the ease of cleanly preparing its surface, making it a model material for the study of surface defects such as grain boundaries.^27^ The HER in sulfuric acid media was selected as the reaction of interest due to its fundamental nature and the fact that this reaction has been frequently studied in previous works *via* SECCM on polycrystalline platinum, focusing on facet effects.^28,29^ Additionally, previous SECCM work in sulfate media has shown unique grain boundary behaviour for some processes on polycrystalline platinum, making it an excellent candidate for a system that may exhibit similar results for the HER.^30^

Some preliminary data on platinum grain boundary HER behaviour is provided in Fig. 1b and c. These data were obtained from a single line of ten SECCM probe landings, providing ten spatially independent linear sweep voltammograms (LSVs) that traverse into potentials that drive the HER. The first five probe landings occurred on a single grain (denoted ‘left grain’), before the sixth landed directly on a grain boundary line, and a further four landings occurred on an adjacent grain (denoted ‘right grain’). This scheme of probe landings is also visible in Fig. 1a. To display these data, the LSVs associated with each grain were respectively averaged and plotted alongside the LSV associated with the grain boundary in Fig. 1b. Clearly presented is the increased current magnitude observed in the grain boundary HER response curve, or equivalently, the more positive onset potential of the HER on this site. An alternate means of presenting these data can be performed by selecting a consistent HER-driving potential (denoted as *V*_0_ in Fig. 1b) from each of the LSVs taken, and plotting the current associated with this potential against the position of the probe (depicted in Fig. 1c). This similarly shows an increase in the detected current associated with the HER on the grain boundary.

Regardless of the means of presentation, the preliminary data shown in Fig. 1 demonstrates the expected response from an ‘active boundary’, wherein some property of the grain boundary appears to cause increased activity for the HER when analysed using SECCM. It is this property of grain boundaries that is the goal of study for this investigation.

### Complexities of using standard SECCM protocols on grain boundaries

Initial surveys of grain boundary activity were performed on a polycrystalline platinum foil (prepared by polishing and annealing; detailed in the Experimental section). To study the local activity of the HER, cyclic voltammetry was employed at a scan rate of 1.2 V s^−1^ to encompass the entire electrochemical window of platinum (including potentials giving rise to the HER). Ultimately, other processes that occur within the electrochemical window of platinum (*i.e.* platinum oxidation, oxide reduction, and the onset of the oxygen evolution reaction) are out of the scope of this study, but limited discussion of the behaviour of platinum oxide reduction is available in the SI, Section S1.

**Fig. 2:**
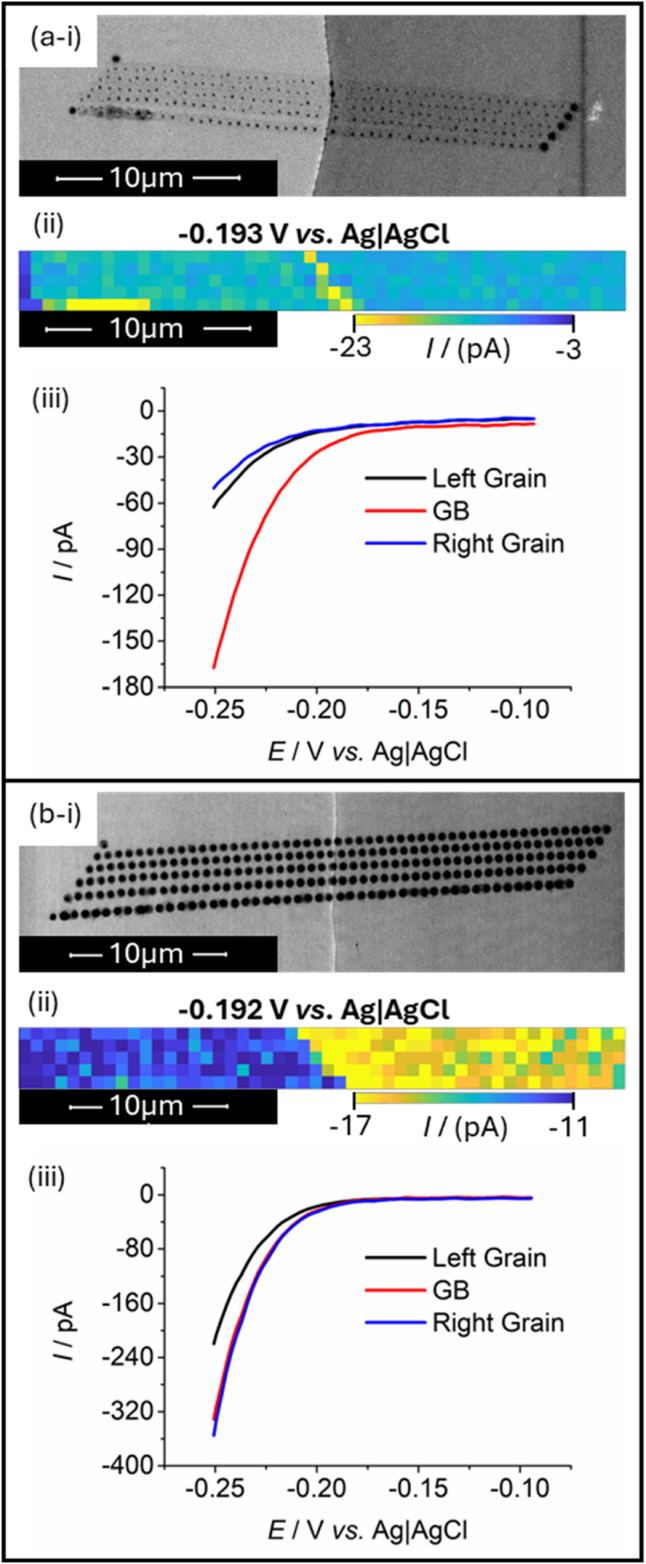
HER response data obtained from two SECCM scans (a) and (b) that included a grain boundary (GB). Provided in each instance is (i) a scanning electron microscope (SEM) image of the scanned area (*i.e.*, taken after the SECCM scan), showing droplet residues left behind by landings of the nanopipette; (ii) an SECCM colour map showing current responses of each probe landing at HER potentials as individual pixels; and (iii) a comparative LSV showing the averaged HER responses of the grain boundary and two adjacent grains. All obtained electrochemical data were acquired using scan rate, *ν* = 1.2 V s^−1^. Scans presented in (a) and (b) were acquired using separately prepared SECCM probes with tip diameters of approximately 200 nm.

SECCM scans were directly positioned to include a grain boundary defect in the approximate centre of the mapped area. The exact grain boundaries selected for analysis were essentially chosen at random, since in each case the boundary of focus was simply in the position that was easiest to manoeuvre the probe towards on the working electrode position of the given day. Generally, small hopping distances (referring to the lateral distance between successive probe landings) of around 500 nm were used to ensure a high likelihood that the grain boundary would be targeted by one of the traversing landings. Two typical scans of this nature are presented in Fig. 2. Note that the parallelogram shapes of the scan profiles (Fig. 2a(i) and b(i)) are not by design, but are instead an artefact caused by drift of the piezoelectric positioners (described in the Experimental section) over the course of a scan. Due to the linear nature of this drift, it is suspected that this is due to thermal effects on the positioning system, though could also arise from any microscale warping or movement of the components in the SECCM setup. Despite this, interpretation of the associated data is not meaningfully impeded.

In taking scans of grain boundaries in this format, it was quickly evident that many grain boundaries appeared significantly more active when performing the HER, when compared to the surrounding grains of the electrocatalyst. One such example is provided in Fig. 2a. In this case, the arrangement of SECCM probe landings is displayed in the scanning electron microscope (SEM) image (Fig. 2a(i)), with some landings providing analysis of the left or right crystalline grains, and some landing directly on the boundary, each being associated with an averaged LSV traversing into potentials that drive the HER. Full analysis of the averaging processes and variability in responses in each case is provided in the SI, Section S2. To clearly show the increased activity that is measured at the grain boundary, an ‘electrochemical activity map’ is provided (Fig. 2a(ii)) wherein an HER-driving potential was selected, and the current at this potential in the LSV of each landing is plotted in a spatially resolved map, with the same dimensions as the layout of physical probe landings. In this case, clearly evident is a higher detected HER activity on the probe landings that match the position of the grain boundary within the scan, shown by the increased current magnitude on these points. Entire LSVs of the average responses of the grains and grain boundary (Fig. 2a(iii)) also clearly indicate the increased measured HER current magnitude on the surface on the grain boundary.

However, this observation was inconsistent, with some boundaries yielding activities significantly higher than the surrounding grains, and some displaying little measurable difference to the adjacent surfaces. One such example is provided in Fig. 2b, wherein neither the activity map nor the averaged LSVs of probe landings (Fig. 2b(ii) and (iii), respectively) showed an increase in activity on the boundary line. No clear increase in current is apparent in Fig. 2b(ii), and the averaged grain boundary LSV in Fig. 2b(iii) appears to lack any significant difference to the averaged LSV obtained from the right grain. From this data alone, it is unclear why the response on the grain boundary matches so closely with the right grain, since the SEM image in Fig. 2b(i) suggests that the boundary-including landings span both the left and right grains approximately equally, and one may expect that it should thus yield a response close to the average of the two. It is possible that some enhancement in activity still exists in this case (though to a lesser extent than is observed in Fig. 2a), or the SEM images taken after scan completion may not accurately represent the true areas contacted by the probe during measurement itself. Nonetheless, this intermittency and high variability in the presence of enhanced grain boundary activity appeared to be functionally identical to observations made in previous SECCM studies of grain boundaries, and warranted detailed investigation.^30–33^

Initially, it was assumed that the measurements of heightened HER activity on some grain boundaries were due to a genuine increase in the intrinsic electron-transfer kinetics, and the inconsistency in the HER activity on these features appeared to indicate a complex relationship between grain boundary structure and catalytic activity, which has been previously observed on other systems using SECCM.^34^ Further study challenged these assumptions, as is discussed in subsequent sections. However, this line of thought is important for contextualising the next stages of investigation.

Under the assumption that there existed a complex relationship between the HER activity and geometric structure of grain boundaries, SECCM measurements of over 50 grain boundaries were taken, similar in form to those shown in Fig. 2. The rationale behind obtaining a large data set in this regard was founded on the inherent complexity of grain boundary structure. A discussion on this is available in the SI, Section S3, detailing the complexity of grain boundaries in terms of their crystallographic geometry, and the extent of analysis required to fully define their structure.

During the analysis of a large array of grain boundaries, inconsistent results were observed regarding HER behaviour in a similar manner to that described in the initial scans. However, close examination of scan areas using SEM revealed conflating effects that appeared to explain the inconsistencies. Instead of activity being correlated with some unknown geometric property of grain boundaries, the HER activity appeared largely dependent on the nature of SECCM probe landings on the grain boundary line. High resolution SEM imagery revealed that grain boundary lines would often cause the wetted area of a given probe landing to stretch along the boundary line, leading to a higher surface area of contact and a proportionally larger current for any given process. It is currently unclear whether the mechanism by which the grain boundaries cause increased wetting is due to an inherently lower surface tension with electrolyte on the defect lines, or if the spreading of electrolyte is due to the ‘ridge-like’ topography of grain boundaries themselves (resulting in behaviour similar to capillary action along the sharp angle of the surface ridge).^35^ Whatever the root cause may be, the vast majority of observed enhancements in HER activity on boundary lines could be qualitatively explained by this phenomenon.

**Fig. 3:**
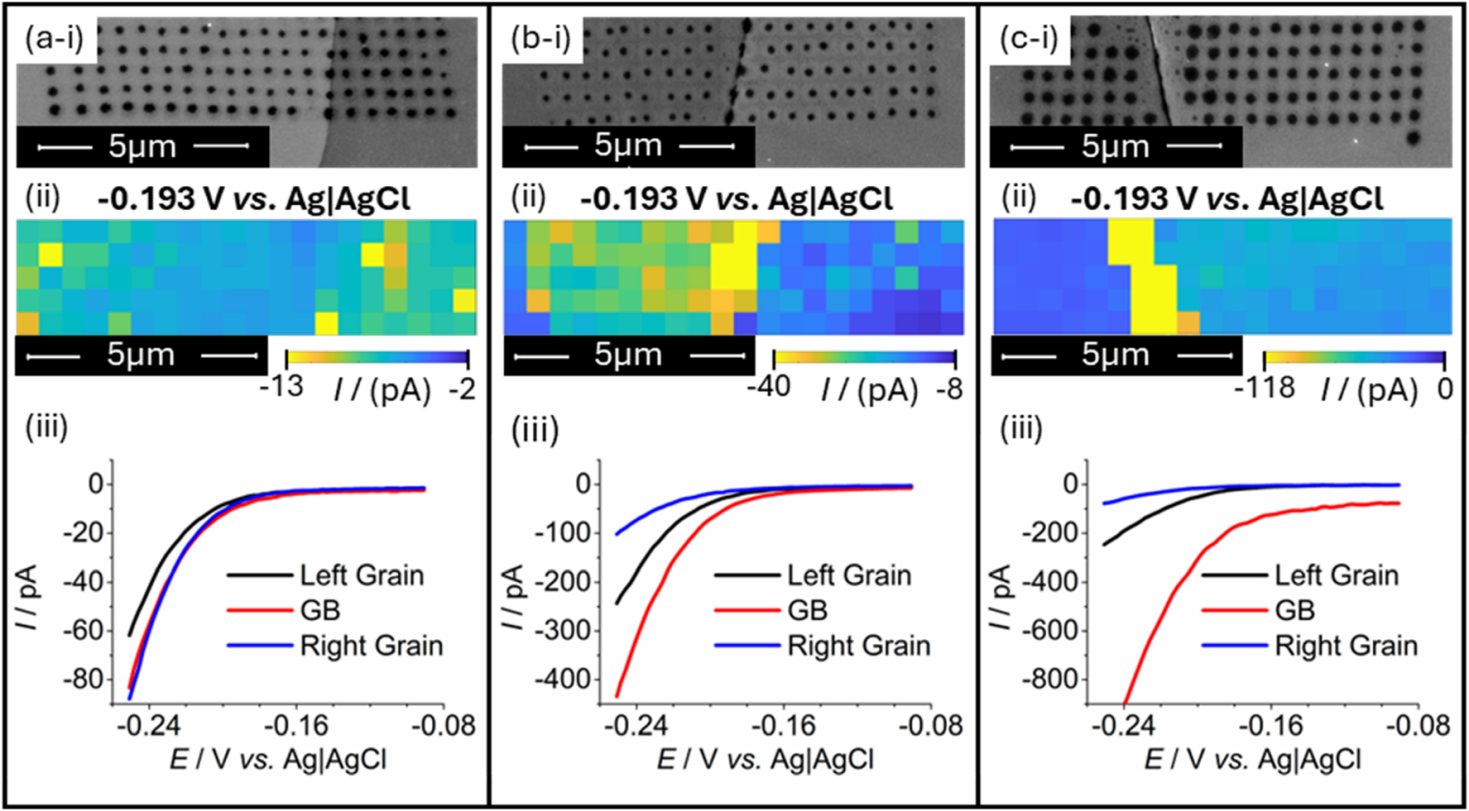
HER response data obtained from three SECCM scans (a)–(c) that included increasingly deformed landing behaviour on grain boundary (GB) lines. Provided in each instance is (i) an SEM image of the scanned area, showing droplet residues left behind by landings of the nanopipette; (ii) an SECCM colour map showing current responses of each probe landing at HER potentials as individual pixels; and (iii) comparative LSVs showing the averaged HER responses of the grain boundary and two adjacent grains. All obtained electrochemical data were acquired using scan rate, *ν* = 1.2 V s^−1^. Scans presented in (a) and (b) were acquired using the same SECCM probe, whilst the probe used for the scan in (c) was prepared separately, all exhibiting tip diameters of approximately 200 nm.

Fig. 3 presents the SEM image, electrochemical activity map (taken at −0.193 V *vs.* Ag|AgCl) and averaged LSVs taken across three grain boundaries exhibiting different meniscus cell wetting behaviour. From Fig. 3, a clear trend in grain boundary HER current can be observed with the associated increased wetting of probe landings. In cases with little to no observed excess wetting on boundary lines (Fig. 3a, showing clearly defined boundary landing residues in Fig. 3a(i)), HER activity is found to be identical to one of the surrounding grains. In particular, no increase in current is observed on the boundary line in Fig. 3a(ii), and the averaged LSV taken from grain boundary measurements in Fig. 3a(iii) appears no different in HER behaviour to the adjacent right grain. With some deformity in the wetted area (Fig. 3b, showing slightly altered boundary landings in Fig. 3b(i)), the HER current is found to partially increase, with Fig. 3b(ii) and (iii) both indicating apparently increased boundary activity through increased HER current magnitude. Note that these situations were also observed in the scans shown in Fig. 2, and this line of reasoning can explain the observed results therein. Additionally, this issue of increased wetting can be extreme in some cases, causing the droplet residues to no longer be identifiable in location or size. Fig. 3c gives a direct example of this, where Fig. 3c(i) shows unclear landing behaviour on the boundary, with electrolyte pooling along the boundary line (visibly evident when comparing to the relatively ‘clean’ boundary in Fig. 3a(i)). In these situations, the measured HER current has an extremely large magnitude compared to the surrounding grains, evident in both Fig. 3c(ii) as a larger magnitude of current at the displayed potential on the grain boundary position and Fig. 3c(iii) where the grain boundary LSV shows HER current to be many times greater than the surrounding material. Furthermore, currents measured in non-faradaic regions of the voltammograms (associated with charging of the capacitive double layer on the working electrode; −0.08 to −0.12 V *vs.* Ag|AgCl, see Fig. 3c(iii)) are visibly many times higher than the surrounding ‘well behaved’ landings.

The correlation between droplet landing area and observed current is not only limited to the HER and the double layer charging region. In scans where the chosen potential range was extended to regions involving the oxygen evolution reaction and platinum oxide formation and reduction, identical trends were observed. The combined correlations suggest that there are extremely strong influences of surface area effects skewing current responses on grain boundary lines.

Whilst the increased wetting of SECCM landings on grain boundaries can be considered interesting behaviour, measurements of this kind are unable to yield information on the intrinsic electrochemical properties of the grain boundary features, since the surface area of landings presents a severely conflating effect on almost all associated data. Instead, techniques must be developed to remove these effects or accurately account for them when analysing electrochemical data. This goal formed the basis of the remainder of this investigation.

### Techniques for the reliable study of grain boundary electrochemistry *via* SECCM

Methods that remove the effects associated with increased droplet wetting on boundaries can be separated into two groups: physical modification of the analytical setup to minimise surface wetting, and data alteration that accounts for the effects of increased contacted surface area where possible. Techniques employed in this study include examples of both.

**Fig. 4:**
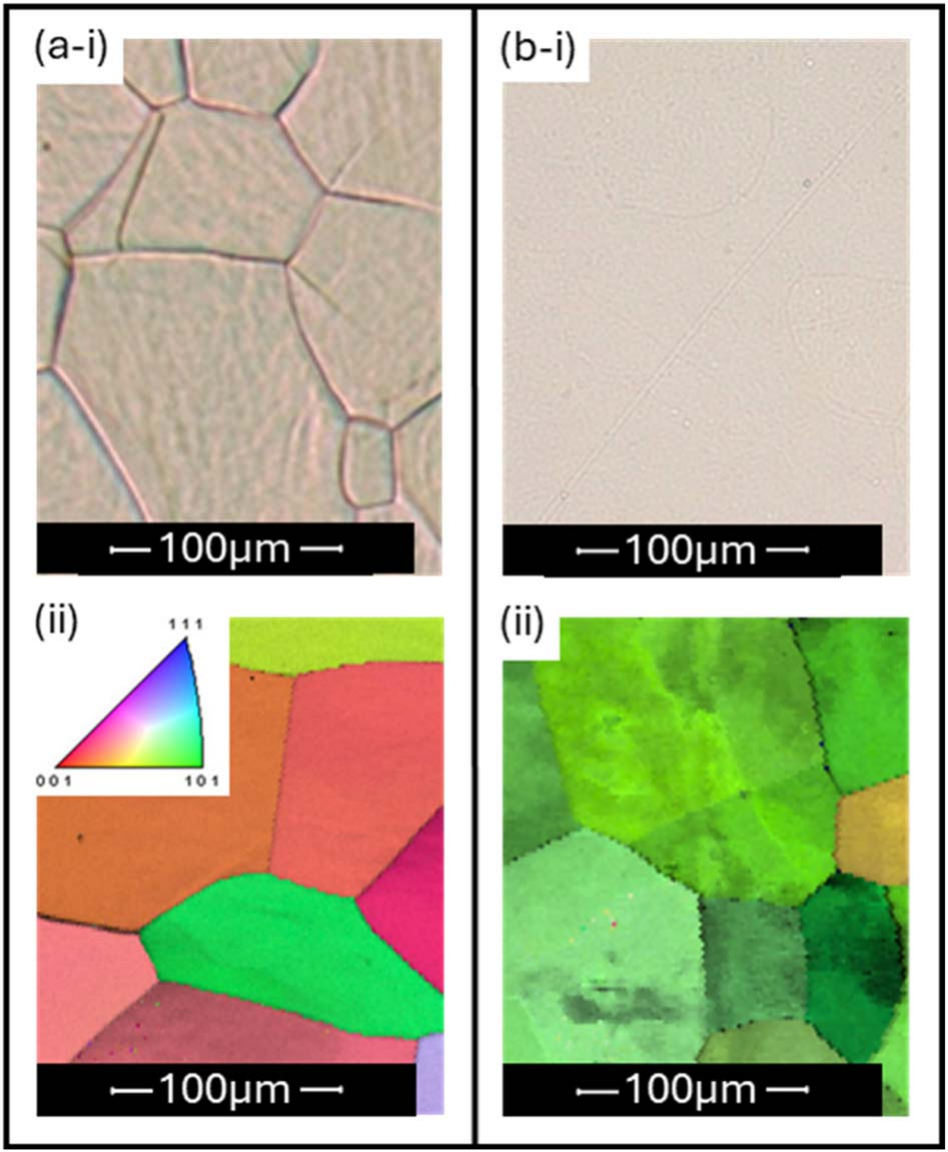
Comparison of two platinum surface preparations, wherein (a) depicts a surface polished and flame annealed many (>30) times, and (b) depicts a platinum surface that had been polished and annealed once without water quenching. Included in each are (i) an optical microscope image of the surface, with grain boundaries visible; and (ii) an EBSD inverse pole figure (IPF) map of the surface, including the IPF colour diagram. Note that the optical microscopy in (i) and EBSD images in (ii) were not taken on the same surface locations, and instead can be considered representative.

To minimise the occurrences of severe surface wetting, changes to the preparation of the polycrystalline platinum surface were made. In the aforementioned tests, platinum foil was repeatedly annealed using a butane flame, followed by quenching the red-hot metal in ultrapure water. This led to the formation of deep-profile grain boundaries that were ideal for observation under an optical microscope (Fig. 4a). However, the ridge-like nature of these features led to the detrimental wetting behaviour discussed previously. Instead, it was found that the quenching step could be neglected during a single annealing process, giving a surface that contained grain boundaries with minimal ridge-like character (Fig. 4b). Slower cooling led to a lower degree of surface deformation of the platinum, making grain boundaries more difficult to see under optical microscopy, but assumed to be less prone to severe wetting effects when performing SECCM. This change in preparation technique had very little effect on the macroscopic electrochemical properties of the platinum surface (see the SI, Section S4), but is ideally suited for keeping the grain boundary topography suitable for SECCM measurement.

Alternate methods of minimising the wetting of electrolyte along grain boundary lines were also considered. Recently, success has been found in the use of electrolyte additives that reduce surface wetting with minimal effect on analysed electrochemical processes.^36^ Additionally, electrochemical cleaning of the platinum surface is known to change its surface properties (possibly altering wetting behaviour), and has been used in past SECCM studies.^37^ Due to the success in modifying the surface preparation presented herein, these alternative routes were not explored in detail, though some discussion of an attempt to simulate electrochemical cleaning on grain boundaries in presented in the SI, Section S5.

With the platinum surface prepared in a manner more suited to SECCM study of grain boundaries, wetting effects as severe as those seen in Fig. 3c were nearly eliminated. However, smaller wetting effects (akin to those observed in Fig. 3b, for instance) were still common upon performing new SECCM scans. Some examples can be observed in the SEM images provided in the SI, Section S6.

With physical alteration of the surface not entirely eliminating surface wetting effects, data alteration techniques were turned to in order to correct for small local increases in landing surface area. Due to these small differences in surface area, the relative activities of each landing cannot be directly compared by their HER currents at a given potential (as can normally be done during SECCM, since each landing is generally considered to have very similar areas of contact in the absence of surface defects).^28^ Instead, comparing local HER activity must be done by comparison of current density, which inherently accounts for surface area of contact.

Producing SECCM maps that measure local current density requires direct measurement of the area of each landing. Ideally, this may be performed by SEM analysis of a scan after it has completed, as the landings leave residue on the surface that can be imaged (such as shown in Fig. 2 and 3). However, given the number of landings contained in a single SECCM scan (ranging from hundreds to tens of thousands), this is a difficult task to implement. Instead, a proxy for surface area can be found in the obtained cyclic voltammograms of each landing in the form of the capacitive double layer charging current, as has previously been reported on porous thin-film materials with SECCM.^38,39^

In the regions of each CV in which no faradaic processes occur (*e.g.*, between 0.15 and 0.45 V *vs.* Ag|AgCl, herein), the current is solely dictated by the rate of build-up of the electric double layer on the working electrode surface.^40^ At a given potential scan rate and electrolyte concentration, this build-up of charge can generally be regarded as being proportional to surface area (since more surface area allows for a proportionally greater extent of charge accumulation).^41^

Given literature estimates on the value of the specific double layer capacitance (*C*_A_) of a platinum surface in 0.5 M H_2_SO_4_,^42,43^ a value of 44 μF cm^−2^ was chosen to perform subsequent calculations (though it should be noted that error in this value should merely lead to an issue in scaling, which is irrelevant in the presented data). Complete calculation of the area (*A*) and current density (*j*) from the double layer charging current (*i*_DL_) and scan rate (*ν*) then becomes a simple conversion described by eqn (1) and (2). It should be noted, however, that on the scale of measurement undertaken, stray capacitance represents a large fraction of the total capacitance measured, and its contribution to *i*_DL_ must be subtracted from the total current measured before the following calculations are performed. In this case, the stray capacitance of the system was measured to be 0.3 pF, as described in the Experimental section, generally contributing to over half of the current observed in the double layer region.1
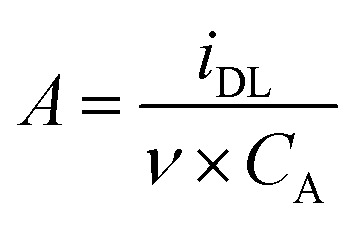
2
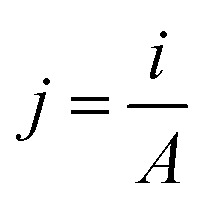


In this way, identifying the double layer charging current associated with each landing allows for the application of a scaling factor such that current densities can be estimated. Importantly, this removes effects associated with probe wetting behaviour on grain boundaries and allows for reliable comparison between local measurements. Complete examples of the methodology and calculations involved with this technique, along with justification on the reliability of this means of estimating surface area of contact, are provided in the SI, Section S7. Additionally, comparisons of this technique against other means of measuring current density are included in Section S8 of the SI, wherein this method is compared to the aforementioned SEM-based analysis of droplet contact area and the estimation of surface area based on charge passed in the underpotential deposition processes of hydrogen. Ultimately, the described procedure involving calculation *via* double layer charging current was deemed the most consistent and most appropriate for this study.

**Fig. 5:**
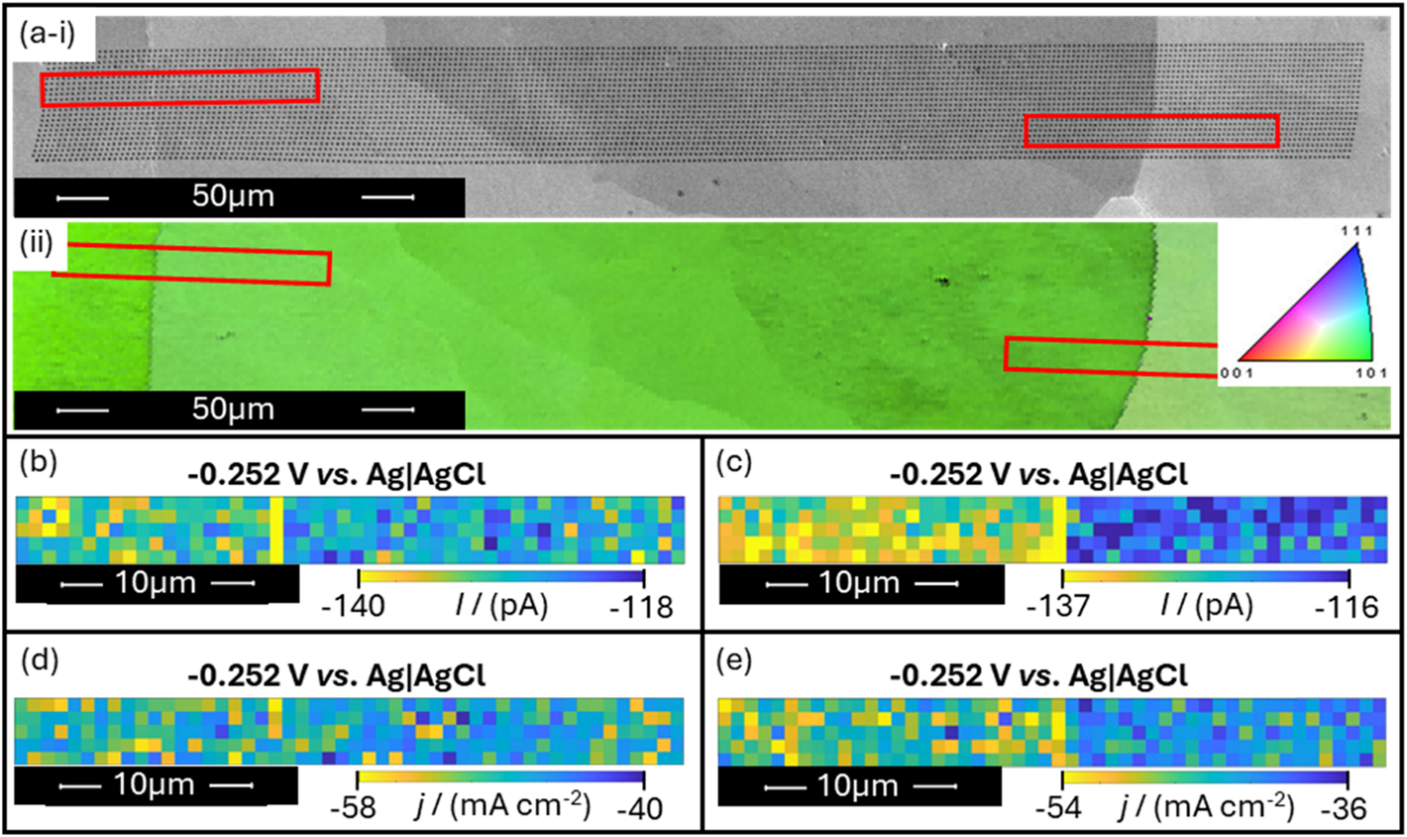
(a) Co-located SEM and EBSD IPF map of an SECCM scan that included 2 grain boundaries (including the associated IPF colour diagram). (b) and (c) present SECCM colour maps of boundary-including regions within the scan in (a), showing current responses of each probe landing at HER potentials as individual pixels. (d) and (e) provide alterations of (b) and (c) respectively by the application of surface area correction procedures, plotting each pixel relative to the calculated current density, not current alone. All obtained electrochemical data were acquired using an SECCM probe with a tip diameter of approximately 250 nm, and a voltammetric scan rate of *ν* = 10 V s^−1^.

**Fig. 6:**
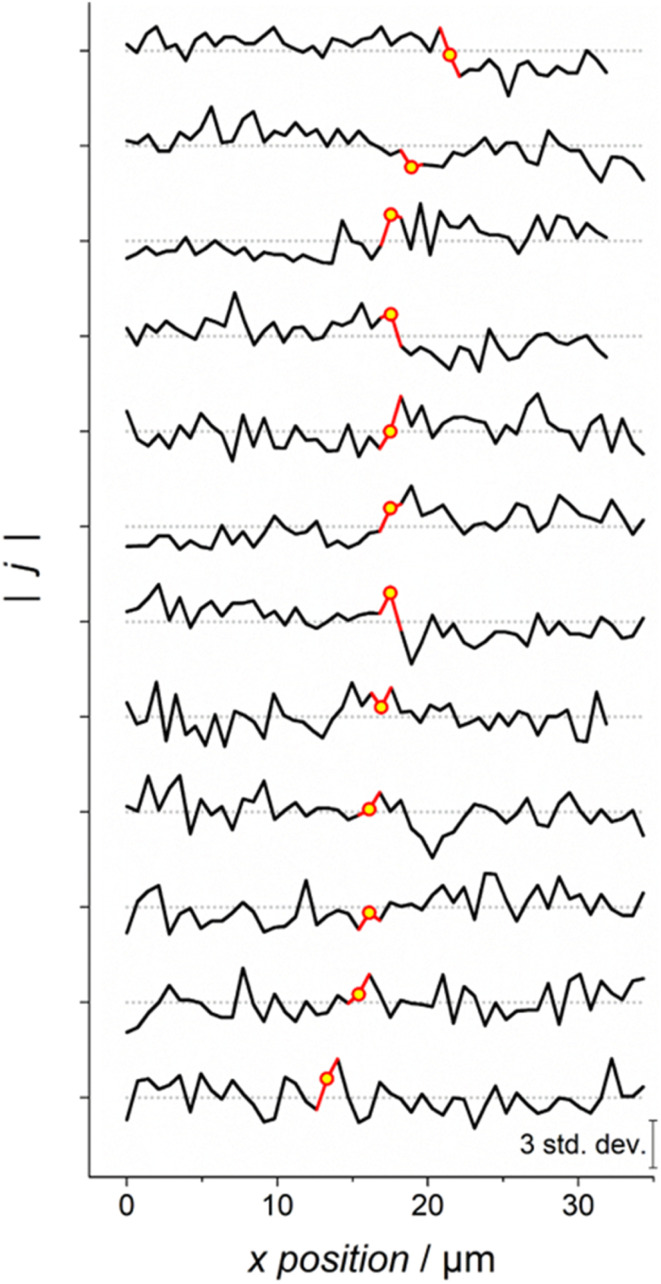
A collection of 12 SECCM scans including grain boundary landings, represented as a direct comparison of HER response current magnitudes at −0.252 V *vs.* Ag|AgCl, comparing responses on a linear landing-by-landing basis. The displayed format matches that already seen in Fig. 1c. Each line is constructed by taking the current density data from a scan similar to those presented in Fig. 5d and e, and averaging the current densities obtained from each vertical column of pixels/landings. These averaged current density values form each line plot, with the value corresponding to the grain boundary landings highlighted in yellow. All current density magnitude values are plotted relative to their *z*-scores against the average current density of the scan (shown as the series of grey dotted lines), wherein the scale of standard deviations against the mean is depicted (bottom right). All obtained electrochemical data were acquired using SECCM probes of approximately 250 nm diameter, and scan rate, *ν* = 10 V s^−1^. Extended analysis of all 12 scans can be found in the SI, Section S6, including a list of which scans were taken using different SECCM probes.

The analysis of current density and the associated removal of locally increased surface wetting can be visualised in Fig. 5. Scans of two grain boundaries show the near-complete removal of the apparent “enhanced activity” observed on the grain boundary when measuring current alone. Note that due to the reduced optical visibility of grain boundaries (shown in Fig. 4b(i)), it became no longer possible to ‘target’ a grain boundary with a given SECCM scan. Instead, very large scans (with dimensions spanning lengths greater than 100 μm) were undertaken in the hope that grain boundaries would randomly be encountered. This is seen in the scans presented in Fig. 5 and 6, wherein only small boundary-including sections of scans are isolated for analysis. A caveat of scanning larger areas also arose in the necessary time to complete each full scan. Applying the same parameters used in the scans shown in Fig. 2 and 3 under these conditions would have led to scans taking over 120 hours to complete, very likely leading to errors or failures before scan completion. To overcome this, it was decided to increase the voltammetric scan rate for the scans shown in Fig. 5 and 6 to 10 V s^−1^. Whilst it is expected that this change does not influence the conclusions taken from these scans, it must be noted that comparisons between later figures and the data shown in Fig. 2 and 3 should be performed semi-quantitatively.

Fig. 5 presents SEM imaging correlated with two different forms of electrochemical activity maps. With SEM and EBSD inverse pole figure (IPF) images of the relevant SECCM scan shown in Fig. 5a, two grain boundary-including regions could be isolated for analysis (highlighted in red). Fig. 5b and c provide standard electrochemical activity maps of those highlighted regions, showing localised HER currents of the probe landings (taken at −0.252 V *vs.* Ag|AgCl). From Fig. 5a–c, the local HER activities of the locations corresponding with the positions of the grain boundary lines appear to be higher than the surrounding surfaces, shown by the higher magnitude of current detected on these locations at the displayed potential. With the suggested method of accounting for surface area effects, Fig. 5d and e are also constructed such that the current of each probe landing is expressed as a current density rather than current alone (calculated by means of eqn (1) and (2)). By applying this surface area correction technique, it is observed that the grain boundary responses are greatly minimised, to the point where it is visually unclear whether the increased HER activity on the grain boundaries is a statistically significant observation.

An observation worth considering when analysing the data in Fig. 5 (and subsequently, in Fig. 6) is the fact that the HER responses of the ‘left’ and ‘right’ grains are generally found to have only small differences. This is a result of the analysed surface prepared by a single annealing cycle exhibiting similar grain orientations—all close to the crystallographic orientation defined as (101) (also seen in Fig. 4b(ii)). It is unclear why this surface was found to predominantly exhibit this orientation, as attempts to replicate this effect have failed on other platinum pieces. It may be that this arises from the original manufacturing of the material, or may be a result of hundreds of annealing cycles slowly allowing the grain surfaces to relax into the (101) orientation. Regardless, the observations of grain boundary behaviour are considered to be unaffected by this property of the studied surface.

The observation of grain boundaries that yield apparent enhanced activity under standard SECCM techniques, but very limited (or possibly non-existent) enhancement when accounting for droplet contact area, presented a somewhat unexpected possibility. It was feasible that all previously observed grain boundary activity for the HER on platinum could be solely attributed to surface area effects. In this case, it would negate the need for finding a structure–activity relationship, since grain boundaries would not be observed to yield enhanced activity when compared to surrounding surface regions on the scale of the relevant SECCM measurements (that is, areas no more than hundreds of nanometres in diameter). Whilst this would not rule out the existence of enhanced grain boundary activity on the atomic scale, it would suggest that catalytic activity for the HER on platinum, from the macroscopic to the scale of hundreds of nanometres, is overwhelmingly dominated by effects other than the presence or density of grain boundaries. Instead, local surface area and crystallographic orientation would be the dominant factors determining local activity. To provide enough evidence to make convincing claims on this, scans were once again taken over a set of 12 grain boundaries, applying the described surface preparation and data correction techniques throughout.

Fig. 6 depicts a simplified analysis of the 12 scans mentioned. Each line graph is constructed such that the *x* axis depicts the lateral position of the probe on the surface, and the *y* axis depicts the relative current densities of HER processes at −0.252 V *vs.* Ag|AgCl for each successive landing. The scale of the *y* axis is constructed such that all points on each line graph are plotted in terms of the number of standard deviations they are from the mean current density obtained across the entire scan (in other words, their *z*-scores). Due to differences in the variability of current density between different scans, and local differences in current density obtained from different scan areas on the working electrode, this normalised approach was found to be the most convenient manner of presenting this set of data, though raw data for each individual scan section can be found in the SI, Section S6, with detailed analysis of all 12 examples.

Every line shown in Fig. 6 is taken by averaging five total linear scans (*i.e.*, each point represents the average current density obtained from five landings sharing the same *x* position, but different *y* positions). In each case, the scans include one vertical grain boundary, meaning that one point in the line should include current contribution from the boundary itself (highlighted in yellow in each case). Looking at these data with the expectation that grain boundaries are inherently active for the HER, one would expect to find increased current density on the highlighted points that are clearly above the average response of the surrounding surface regions, similar to that observed in Fig. 1c.

By closely examining the 12 scan depictions in Fig. 6, it is clear that increased activity is not found on the studied grain boundaries. Instead, it appears that the locations corresponding to grain boundaries do not yield current densities that are significantly different to the surrounding material. When compared to the surrounding 49 positions, none of the grain boundaries even represent the most active site analysed in its respective scan. Having 12 examples available, with none showing significant intrinsic activity of the studied boundary line, should serve as clear evidence that grain boundaries showing high HER activity under these conditions are a rare phenomenon at the very least.

These results appear to provide strong evidence supporting the aforementioned notion that grain boundary HER activity on platinum is not enhanced to a significant degree, at least on the scale of the performed measurements (*i.e.*, nm–μm scale). The previously observed activity using standard SECCM techniques may thus be an artefact of how these scans are performed. A logical conclusion of this result is that heightened platinum grain boundary activity is actually a misconception with regard to the HER, or possibly that this heightened reaction rate only occurs on a very low proportion of boundaries.

In making these claims regarding the lack of enhanced grain boundary HER activity on platinum, care must be taken when considering any subsequent conclusions. The observed result may only apply to the grain boundaries exhibited by the platinum surface under the conditions of preparation described. It may be the case that by intentionally favouring the production of boundaries of low ‘ridge-like’ character, bias is being introduced into the system such that boundaries with electrochemically unique properties can no longer form, despite possibly still being present in most samples of the polycrystalline metal. Certainly, the consistency in surface orientation suggested by Fig. 4b(ii) may indicate lack of variability in surface structure, possibly extending to boundary structure also.

An additional caveat that applies to the conclusions of this study arises from the means of correcting for current density. As described previously, the HER current density at a given potential is estimated using the contacted surface area suggested by the double layer capacitance of the working electrode surface. Whilst this capacitance is generally assumed to depend solely on surface area, some evidence suggests that it is slightly influenced by surface structure.^44^ Additionally, single crystal data on platinum surfaces has shown that the adsorption of OH species makes significant contribution in the region selected to be the nominal double layer charging region on some surface structures in sulfuric acid media.^45^ In the case of either of these effects being significant, this would suggest that estimates of double layer capacitance and subsequent surface area calculations are not truly consistent. If the HER activity and double layer capacitance of grain boundary defects are somewhat correlated, or if processes occurring on the nominal double layer charging region are overexpressed on grain boundary sites, this method would lead to false negatives. This is considered unlikely, since all double layer regions encountered in this study appeared clearly defined with very little dependence on surface structure, and there was no indication of the double layer capacitance and HER activity being correlated between different grain orientations (data not shown). Whilst not expected to be relevant here, inconsistency in double layer capacitance represents a possible limitation of this technique when studying similar surfaces in this manner.

Being unable to observe significant increases of activity at grain boundaries on the scale of the employed SECCM setup can also be backed up by logical assumptions. If one first accepts the notion that a grain boundary defect has a functional width of only a few atomic distances (less than 2 nm),^46^ and the proposed electrochemical ‘active zone’ has a similar profile, it follows that such a grain boundary footprint would take up 1% of the area of a typical SECCM probe landing performed in this study. Hence, to meaningfully increase the current response on this location, such a grain boundary must be at least 10 times more active than the surrounding material to yield even a 10% increase in current response (calculation details for these values are provided in the SI, Sections S9 and S10). Whilst not impossible, this level of increase in activity is even greater when working macroscopically, and would be very surprising to observe.

**Fig. 7:**
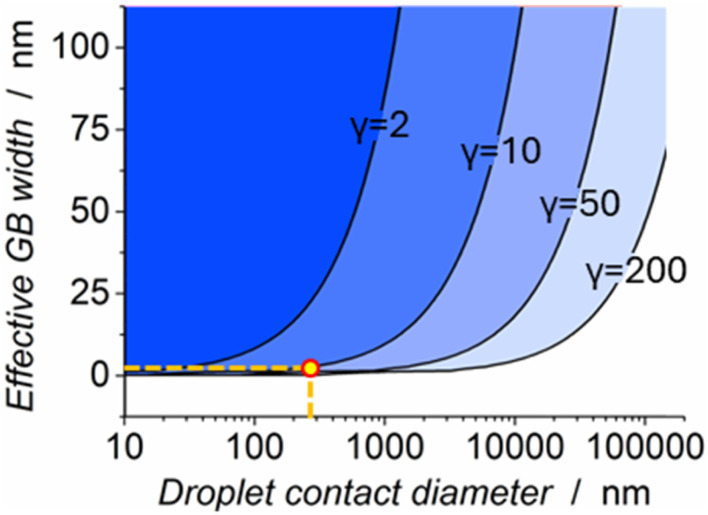
Graphical depiction of the conditions necessary for grain boundary (GB) activity to be detected by SECCM. The *x* and *y* axes are defined by parameters of a given experiment: respectively, the width of SECCM probe landing droplets and the effective electrochemically active width of the grain boundaries being studied. The coloured regions are ‘zones’ in which grain boundary activity can be detected (by the advent of a 10% increase in current density on landings that include a grain boundary). The parameter ‘*γ*’ defines the ratio of current density on the active grain boundary to that of the bulk material. The droplet contact diameter relevant to this study (approx. 250 nm) is highlighted with a vertical dotted line, and the expected grain boundary active width of 2 nm is highlighted with a horizontal dotted line. The highlighted point denotes the expected approximate parameters of the studies in this work.

When considering the limitations of detecting any enhanced activity on a grain boundary, or indeed, any nanoscale surface feature, it is useful to formalise the parameters necessary for a given feature to be resolved by SECCM. An attempt is made to graphically depict the limitations involved in detecting grain boundary activity in Fig. 7, which attempts to define approximate conditions (diameter of droplet contact and the electrochemically active width of a grain boundary) that must be met for a boundary-including probe landing to show a 10% increase in current density when compared to surrounding material. Coloured regions in the graph represent conditions where grain boundary activity is, in this way, detectable by SECCM. Each region border is defined by *γ*, which represents the ratio of the current density on the active region of the grain boundary to the current density of the surrounding bulk surface. For example, when working with a probe that yields droplet landing diameters of 1000 nm, and grain boundaries that exhibit an electrochemical ‘active zone’ of 25 nm width, their activity could be detected provided this *γ* parameter is at least 2–10. In detail, the graphic does not suggest means of accurately determining individual experimental parameters such as the active width of a grain boundary or the diameter of droplet contact, but instead suggests the necessary approximate value of *γ* such that the SECCM detection of enhanced grain boundary activity is feasible if these parameters are already known.

In this study, droplet contact diameters are on the order of 250 nm, and it is assumed that the ‘active zone’ of a grain boundary is likely to be 5 nm or less in width. These parameters are directly highlighted in Fig. 7 itself. As depicted, this study (and the results in Fig. 6) could only expect to find evidence of grain boundary activity if *γ* is greater than 10–50. In general, parameters lying towards the upper left of the graph are capable of detecting very low levels of grain boundary activity (down to *γ* < 2), whilst this detection becomes increasingly difficult towards the bottom right, reaching regions where only extremely high levels of relative activity (*γ* > 200) are detectable. The full derivation of the effect of droplet diameter, grain boundary width, and *γ* on the detectability of grain boundary activity is performed and discussed in the SI, Sections S9 and S10.

Fig. 7 serves to suggest that ideally, grain boundary features of the suspected width would be more appropriately measured by SECCM using smaller probes. This is visualised by moving the highlighted point to the left, entering regions where enhanced grain boundary activity could be detected at lower values of *γ*. Note that the probes used herein (∼250 nm diameter) are among the smallest used for SECCM imaging,^25^ and were the finest that were able to be reliably manufactured from borosilicate capillaries on the heating coil-based gravity puller and laser puller device employed herein (further details available in the Experimental section).

Alternatively, other studies suggest the existence of an electrochemical ‘footprint’ of a grain boundary for some processes, wherein the electrochemical ‘active zone’ of the defect extends much further than the atomic misalignment of the adjacent grains. This active zone has been observed to exceed a width of 2 μm in some cases.^33,47^ This phenomenon would have allowed boundaries to be much more feasible to observe in SECCM data during this study, with activity possibly extending laterally across multiple probe landings. For the HER on polycrystalline platinum however, this effect was not observed.

Whilst the apparent lack of abnormal activity of platinum grain boundaries for the HER is certainly not applicable to all other reactions and all other surfaces (corrosion of steel and copper, for instance, are processes where grain boundaries have well-established influence),^13,14^ this study shows that there exist processes where grain boundaries do not confer enhanced activity to electrocatalyst behaviour. It is suggested that current and future SECCM studies ensure the type of scrutiny undertaken in this investigation when making claims on the local activity of grain boundaries. This is particularly true when observed activity increases are small enough that they can be accounted for by effects arising from the surface area of contact.

## Conclusions

The intrinsic properties of grain boundaries on polycrystalline electrocatalyst surfaces have long been a subject of interest when it comes to the design of an efficient electrode material. In many cases, evidence exists to support the notion that the atomic disorder inherent to the structure of a grain boundary yields an enhancement in electron transfer for inner-sphere or electrocatalytic processes. As a result, a question arises over whether this is a universal phenomenon across all electrocatalysts that exhibit polycrystalline structure, which calls for a convenient means of directly studying the electrochemical properties of individual grain boundaries.

In this work, SECCM is used as a suitable tool for the direct analysis of single grain boundaries on a polycrystalline platinum working electrode surface. Due to its ability to isolate small areas of the working electrode (in this case, areas with a diameter on the order of hundreds of nanometres), this technique was found to be well suited for the direct analysis of the acidic HER on these features. By the combined SECCM and SEM analysis of several unique grain boundaries, it was first found that surface tension effects on grain boundary lines often produce undesirable electrolyte wetting when using droplet-based techniques such as SECCM, causing many boundaries to exhibit falsely enhanced apparent activity due to the increased surface area of electrode wetting.

By careful control of annealing processes in the preparation of platinum and the estimation of electrochemical surface area from charging the double layer on the platinum surface, it was found that the effects of increased surface wetting could be effectively eliminated. This allowed for direct measurement of current density when analysing grain boundaries. Through the use of these techniques on a total of 12 unique platinum grain boundaries, it was found that all examples gave no significant increase in activity for the acidic HER when working on the scale of hundreds of nanometres. This suggests that grain boundaries are not inherently more active in all polycrystalline electrocatalysts and across all processes, but that there are instead some situations in which they do not meaningfully contribute an enhancement to overall electrocatalyst behaviour.

The studies in this work provide a convenient means of direct grain boundary analysis for electrocatalyst materials, particularly for the avoidance of false-positive results that can be attributed to surface tension effects alone. This type of analysis will enable the conclusive determination of grain boundary effects in electrode processes, and in cases where grain boundary activity is meaningful, will allow for quantitative measurements of their properties. In future studies that may choose to fully characterise the crystallographic geometry of grain boundaries and correlate this to electrochemical data, it is suggested that these techniques will be an invaluable tool in determining structure–activity relationships.

## Experimental

### Preparation of electrolyte

All sulfuric acid electrolytes were prepared at a concentration of 0.5 M, sourced from Sigma-Aldrich, ACS Reagent, 95–98%. Dilution was performed using deionised water (Direct-Q Water Purification System, Milli-Q, USA). Stock solutions were stored in airtight glass vials prior to use.

### Preparation of platinum surface

The analysed platinum surfaces were prepared from pieces of smooth platinum foil (99.95%). Two platinum samples were utilised during this investigation, the first of which exhibited 0.5 mm thickness, and a size of 10 mm by 7 mm (Goodfellow, U.K.) – used to produce the scans shown in Fig. 2, 5, and 6. The second sample was a 1 g platinum bullion bar, giving a thickness of 1 mm, and a size of 7 mm by 7 mm (Argor-Heraeus, Switzerland), used to produce the scans shown in Fig. 3. Prior to SECCM analysis, the surface of platinum was sanded flat, polished using a 9 μm diamond polish (M23-9, ProSciTech, Australia), followed by a 1 μm diamond polish (M23-1, ProSciTech, Australia), and subsequently annealed using a butane flame for 10 seconds. Following this annealing, initial investigations (shown in Fig. 2 and 3) involved the immediate quenching of the platinum sample in deionised water (Direct-Q Water Purification System, Milli-Q, USA). However, later investigations (shown in Fig. 5 and 6) avoided the quenching step by instead allowing the sample to cool in ambient air. These procedures of flame annealing the platinum foil were selected in the attempt to be broadly representative of how one may prepare a platinum working electrode in everyday use for voltammetry.

### Preparation of reference and quasi-reference counter electrodes

The Ag|AgCl QRCE was in the form of a silver wire (Goodfellow, UK, 0.125 mm thickness, 99.99%), anodised in saturated potassium chloride using a 9-volt battery for 30 seconds to produce a surface coating of AgCl. QRCEs produced in this manner have previously been shown to possess a stable potential on the minutes to hours timescale (∼1 mV drift) under acidic conditions in SECCM.^48^ Between scans, the Ag|AgCl wire was stored in 0.5 M H_2_SO_4_. To calibrate the potential of this electrode in the applied solution, the open circuit voltage of the QRCE was measured in 0.5 M H_2_SO_4_ against a leakless miniature Ag|AgCl reference electrode (3.4 M KCl, eDAQ, Australia).

In scans depicted in Fig. 5 and 6, the use of a QRCE was abandoned in favour of the direct usage of the described leakless miniature Ag|AgCl reference electrode as a counter-reference electrode. This was done since the reference electrode was found to exhibit even higher stability in potential than the QRCEs used. Its use ensured extremely minimal potential drift, such that any observed trends could be considered more reliable. Additionally, it had the benefit of removing the need to adjust any reported potentials, since all processes were inherently measured against the silver chloride electrode under this regime. Full justification of the stability of the potential of this electrode under this kind of use is provided in the SI, Section S11.

It should be noted that all potentials referred to in this paper are quoted with reference to the miniature Ag|AgCl reference electrode. In cases where a silver wire QRCE is used, potentials are always adjusted accordingly such that values are given against the miniature Ag|AgCl reference.

### SECCM setup

The nanopipettes used in each SECCM measurement were prepared *via* two methods. Scans in Fig. 2 and 3 were taken using probes prepared from borosilicate capillary tubes (BF100-50-10 Sutter Instruments, USA, 1.0 mm OD, 0.5 mm ID, 100 mm length). To pull these capillaries into nanopipettes, a capillary gravity-puller (PC-100, NARISHIGE Group, Japan) was used to produce tips of 200 nm diameter. A single pulling stage was implemented by setting heat 63, with maximum weight.

In the later stages of this investigation, 250 nm diameter nanopipettes were prepared from identical glass capillaries, using a laser puller device (Model P-2000, Sutter Instrument, USA). A two-stage program was utilised, the first utilising settings of heat, 270; filament, 3; velocity, 25; delay, 150; pull, 0. The second stage used heat, 240; filament, 2; velocity, 23; delay, 150; pull, 170.

Following the production of these nanopipettes, they were filled with electrolyte solution described above. With filled probes, scans in Fig. 2 and 3 simply inserted a QRCE prior to SECCM measurements. In later scans (Fig. 5 and 6), a leakless miniature Ag|AgCl reference electrode (eDAQ, Australia) was used directly as a reference-counter electrode. This was achieved by attaching a plastic pipette tip (T-200-Y, Axygen Scientific, USA) to the upper end of the glass probe *via* a friction fit wrapped in parafilm, filling this space with more electrolyte, and inserting the reference electrode directly. This technique is based on previous work,^37^ and proved extremely convenient when using any counter-reference electrode with a profile too large to fit inside a capillary of 0.5 mm inner diameter.

To perform SECCM measurements, a scanning electrochemical probe microscope was utilised, as previously reported.^49^ The main component of this device was a 3-axis piezoelectric positioner (Nano-3D200, MadCityLabs, USA, 200 × 200 × 200 μm range), used to manoeuvre a prepared nanopipette. Fine manual control of the overall nanopipette position (performed to locate the system's position above the sample surface) was achieved with a 3-axis micropositioner (9064-XYZ-M, Newport, USA).

The macroscopic working electrode (*i.e.*, polished polycrystalline platinum) was attached to a large SEM stub using spring clips (GTP16144-9-20, ProSciTech, Australia) to maintain good electrical connection. This electrode assembly was mounted in an unsealed 100 mL plastic container (HPL931, Lock&Lock, South Korea), allowing the use of a flow of humidified nitrogen gas through a gas inlet port (Omnifit Connector, Kinesis, UK) to maintain an oxygen-free atmosphere for the experimental measurements (supplied at a rate of approximately 0.1 L min^−1^). To maintain a fully humidified atmosphere, the nitrogen gas was first passed through a bubbler of distilled water. Three circular holes were included in the container lid – a central hole (∼10 mm diameter) to allow the nanopipette probe to approach the working electrode surface, and two holes either side (∼20 mm diameter, replaced with glass) in order to both supply light to the surface when required, and to give a clear view from an optical camera (Axiocam ERc5s, ZEISS, Germany; with magnification lens 44 mm/3.00× InfiniStix, Infinity, USA).

The described setup, including the camera, nitrogen-purged environment cell, and pipette positioner, was constructed on top of a vibration isolation stage (25BM-8, Minus K Technology, USA) to ensure minimal effect of external vibration on the pipette positioning. Additionally, to eliminate external electromagnetic effects on current measurements, the entire apparatus was placed inside a closed Faraday cage (Monash Instrumentation Facility, Australia), including an internal lining of acoustic insulation foam (Adhesive PUR Foam, RS PRO, UK).

To perform each SECCM measurement, electrical potentials were applied to the QRCE or reference-counter electrode (depending on which was used) *via* a programmed system made with the use of LabVIEW (National Instruments, USA). A link to the working electrode acted as the ground connection of the circuit. The ground connection was itself made through a variable-gain low-noise current amplifier (DLPCA-200, FEMTO, Germany), allowing sensitive detection of current down to the picoamp scale.

All SECCM data were obtained *via* voltammetric hopping mode, in which the pipette probe was first manually positioned above a region of interest, the Faraday cage closed, and then the probe repeatedly approached to the electrode surface (at variable rates, on the order of 1 μm s^−1^) with an applied potential of approximately 1.25 V *vs.* Ag|AgCl during the approach. Upon landing on the surface, surface-meniscus contact was detected by the induced double layer charging current, which exceeded approximately 2 pA on the current amplifier. With landing detected, the probe movement was halted, and a voltammetric scan was performed (by applying varying potentials to the system – once again programmed through the LabVIEW software, itself based on the Warwick Electrochemical Scanning Probe Microscopy (WEC-SPM) platform, available from: http://warwick.ac.uk/fac/sci/chemistry/research/electrochemistry/wec-spm/).

Scan rates of the performed voltammograms were varied between different experiments over the course of the investigation, so exact details are provided in relevant figures. During any given voltammetric scan, current measurements were taken every 2 μs and averaged 256 times, giving a data acquisition rate of approximately 514 μs (2 × (256 + 1) μs) per data point. This data acquisition was performed by an FPGA card (NI USB-7855R, National Instruments, USA), which directly processed the signal from the current amplifier for collection by the LabVIEW software. Next, the voltammogram profile setup involved an initial potential of 0.5 V (*vs.* Ag|AgCl), first sweeping to a reducing potential limit of −0.28 V, before raising to an oxidising potential limit of 1.80 V and returning to the initial potential.

After each voltammetric scan, the probe was retracted and repositioned at a new location in a repeating pattern to generate a grid of data (with varying dimensions), each point being separated by 0.5–0.7 μm.

In referring to all cases where surface area of droplet contact was estimated and utilised in data processing, it is important to note that raw measurements of double layer charging currents were subject to stray capacitance (*C*_stray_) that needed to be accounted for in calculation (as described in the SI, Section S7). In this case, *C*_stray_ was measured by performing voltammetric cycling when the SECCM probe was not in contact with the surface, yielding a value of *C*_stray_ = 0.3 pF.

All raw voltammetric data were processed using MATLAB software (MathWorks, USA). To generate SECCM colour maps, current measurements at a particular applied potential were represented as a colour scale, with each pixel of the video representing a unique voltammogram spatial position (arranged in the same way that the physical scan was taken). Other data processing, including the display and averaging of voltammograms, alongside all current comparisons, were performed with Excel software (Microsoft, USA).

### Complimentary surface analysis

Optical visualisation of all scan areas was taken using an Axiolab 5 microscope (ZEISS, Germany). To obtain digital images, an Axiocam 105 color (ZEISS, Germany) optical camera was attached to the described microscope and used for this purpose. With the observed difficulty in optically analysing scan areas on the scale of micrometres, a scanning electron microscope (FEI Quanta FEGSEM) was utilised for accurate measurements of the shape, positioning, interaction with crystal facets, and area in each scan.

Additionally, the determination of crystal facet orientation was performed using EBSD techniques on the same SEM instrument. A TSL Hikari detector at an accelerating voltage of 20 kV was utilised for this, with samples tilted 70° relative to the detector. These EBSD data were processed using TEAM EDS (EDAX, USA) to yield images in the inverse pole figure colour map format.

## Author contributions

HBS and CLB provided experiment conceptualisation. HBS conducted the data curation, formal analysis, investigation, and writing. LFG and AH conducted data curation. CLB provided supervision.

## Conflicts of interest

The authors declare that they have no known competing financial interests or personal relationships that could have appeared to influence the work reported in this paper.

## Supplementary Material

SC-016-D5SC03829D-s001

## Data Availability

Data will be made available upon reasonable request to the corresponding author. Supplementary information: analysis of grain boundary activity for platinum oxide reduction (Section S1); demonstration of averaging processes utilised for figures in the main text (Section S2); discussion of the complexities in the determination of grain boundary geometry (Section S3); a macroscopic comparison of surface preparation techniques used in this study (Section S4); simulation of electrochemical surface cleaning in SECCM scans of grain boundaries using multiple voltammetric cycles (Section S5); extended data analysis on scans shown in Fig. 6 (Section S6); demonstration of the calculation of CDL and its use in scaling data to the electrochemical surface area of contact (Section S7); attempts at using alternative techniques for scaling data to the electrochemical surface area of contact (Section S8); calculation of expected percentages of SECCM droplet areas taken up by a grain boundary (Section S9); estimation of parameters for realistic detectability of grain boundaries by SECCM (Section S10); and use of a leakless Ag|AgCl reference electrode as a counter-reference electrode in a two-electrode system (Section S11). See DOI: https://doi.org/10.1039/d5sc03829d.
