# Supplementary material for: Intrinsic electrocatalytic activity of platinum grain boundaries: correcting measurement artefacts in scanning electrochemical cell microscopy (SECCM)
Source: Chem Sci. 2025 Sep 10;16(42):19929–43. doi: 10.1039/d5sc03829d (PMC12477635; doi:10.1039/d5sc03829d)
Supplement: SC-016-D5SC03829D-s001 [file SC-016-D5SC03829D-s001.pdf]

Supplementary Information (SI) for Chemical Science.
This journal is © The Royal Society of Chemistry 2025

Supporting Information for:

Intrinsic Electrocatalytic Activity of Platinum Grain Boundaries: Correcting Measurement Artefacts in Scanning Electrochemical Cell Microscopy (SECCM)

Harry B. Swan, Lachlan F. Gaudin, Alannah J. Hunt and Cameron L. Bentley*

School of Chemistry, Monash University, Clayton, 3800 VIC, Australia

***Corresponding author**: cameron.bentley@monash.edu

Contents

Section S1. Analysis of grain boundary activity for platinum oxide reduction......................................3

Section S2. Demonstration of averaging processes utilised for figures in the main text 5

Section S3. Discussion of the complexities in the determination of grain boundary geometry..............6

Section S4. A macroscopic comparison of surface preparation techniques used in this study 8

Section S5. Simulation of electrochemical surface cleaning in SECCM scans of grain boundaries using multiple voltammetric cycles 9

Section S6. Extended data analysis on scans shown in Figure 6..11

Section S7. Demonstration of the calculation of CDL and its use in scaling data to the electrochemical surface area of contact 17

Section S8. Attempts at using alternative techniques for scaling data to the electrochemical surface area of contact...21

Section S9. Calculation of expected percentages of SECCM droplet areas taken up by a grain boundary 26

Section S10. Estimation of parameters for realistic detectability of grain boundaries by SECCM 28

Section S11. Use of a leakless Ag|AgCl reference electrode as a counter-reference electrode in a two-electrode system 30

References 31

Section S1 – Analysis of grain boundary activity for platinum oxide reduction

Whilst the majority of this work was performed in reference to the acidic hydrogen evolution reaction, the same techniques of grain boundary analysis can be applied to other processes on the platinum working electrode surface. In the voltammograms undertaken, effort was made to cycle the platinum surface through surface oxidation to form PtO_x, and subsequent reduction of this oxide layer (though this is not presented in any data within the main text). The platinum oxide reduction (POR) process in particular can be analysed for grain boundary activity in the same manner as the HER, and this is depicted in Figure S1-1.

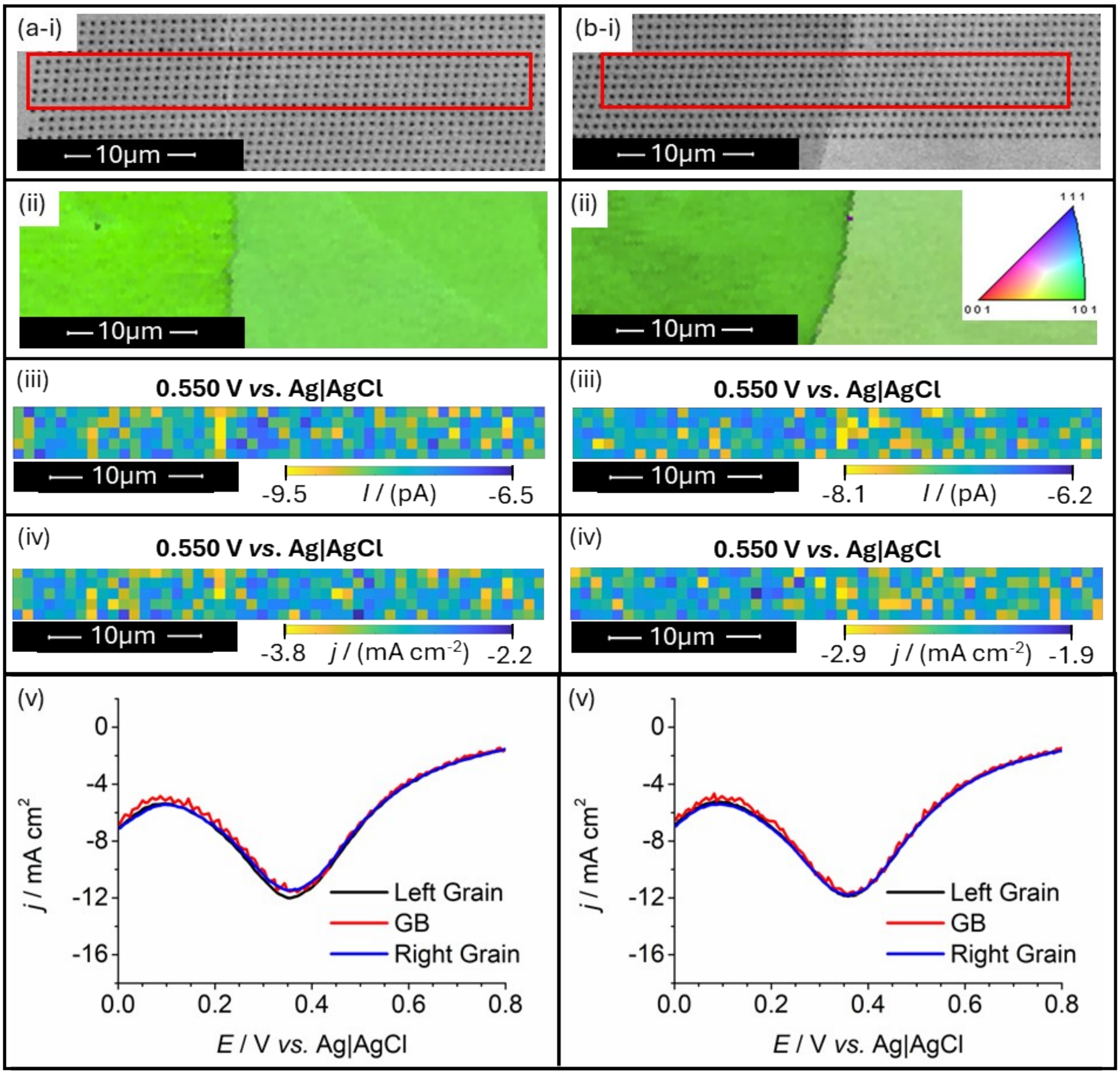

Figure S1-1: Extended analysis of two (a), (b) grain boundaries. In this case, analysis relates to the POR process. Images show (i) an SEM image with the scanned region highlighted; (ii) a co-located EBSD image of the scanned area; (iii) an SECCM colour map of the GB-including scan region, showing current responses of each probe landing at platinum oxide-reducing potentials (0.550 V vs. Ag|AgCl) as individual pixels; (iv) an alteration of the SECCM colour map made by applying surface area correction described in the main text, plotting each pixel with colours relating to current density instead of current alone; and (v) a comparative LSV showing the averaged POR responses of the grain boundary and two adjacent grains, obtained on the negative sweep of the voltammogram.

Figures S1-1a and S1-1b depict two SECCM scan regions that each include a grain boundary. In each case, (i) shows an SEM image taken after the scan was completed, with the analysed region highlighted in red. (ii) then provides conclusive evidence of the presence of a grain boundary with an EBSD image. (iii) shows the SECCM colour map displaying the obtained current obtained at a POR-driving potential on the reduction sweep (0.550 V vs. Ag|AgCl). In both cases, these current maps show some evidence of an increase in the current obtained on the grain boundary, when compared to the surrounding regions. However, accounting for surface area using Equations 1 and 2 of the main text yields the current density maps provided in (iv), and the voltammograms in (v), depicting no significant increase in intrinsic activity on the boundary lines. This is identical to the trends found when analysing the HER.

Section S2 – Demonstration of averaging processes utilised for figures in the main text

Figures throughout the main text and Supporting Information sections of this work make significant use of averaging voltammetric responses from various SECCM probe landings within a scan. In using this representation of voltammetric data, the variability in responses obtained within regions that are expected to be homogenous is not directly clear. Hence, this section serves to demonstrate typical variabilities when performing the mentioned averaging processes.

The scan section processed and shown in Figure 2a in the main text (with averaged voltammetric data directly presented in Figure 2a-iii) is used as an example, shown in Figure S2-1. In this case, low variability is observed for each of the three regions (denoted left grain, grain boundary, and right grain) where averages have been taken. This result is considered representative of the voltammetric data shown throughout the main text, wherein the relative standard deviations of current values obtained from successive landings on equivalent regions are generally less than 10%.

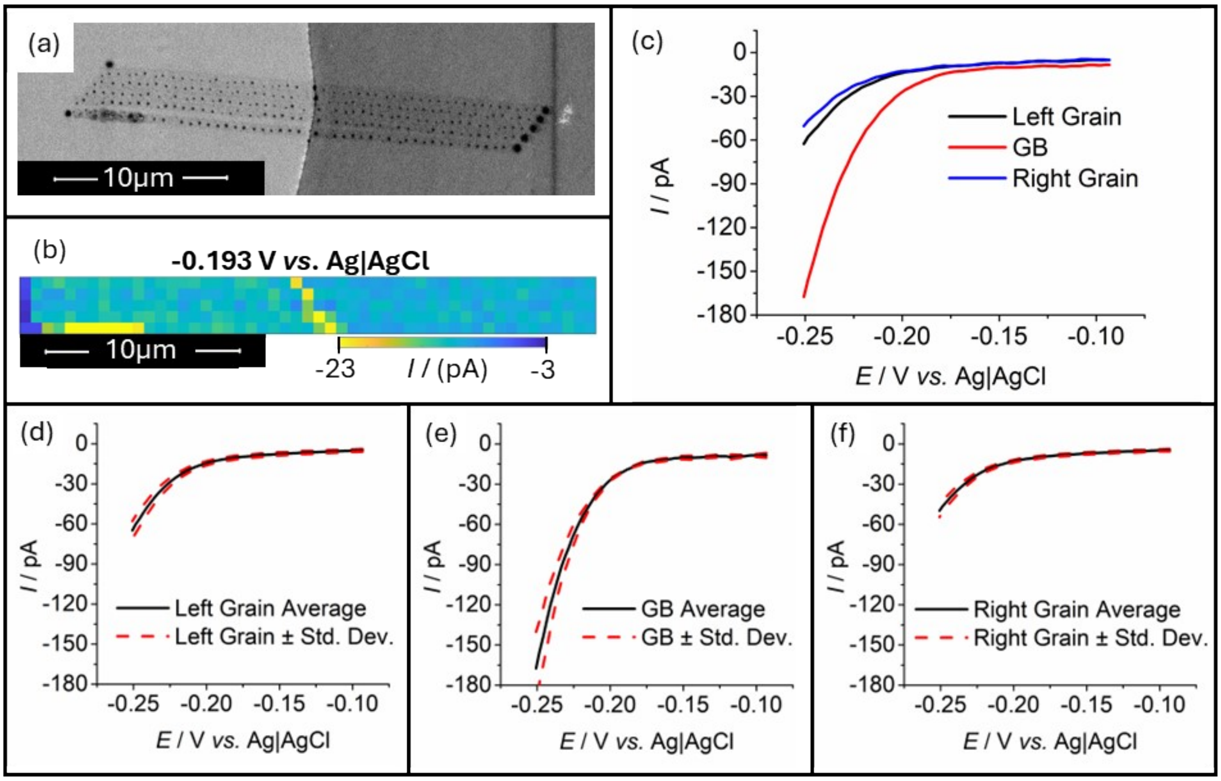

Figure S2-1: Demonstration of the variability in voltammetric responses of SECCM probe landings on the three areas of interest (left grain, GB, right grain) in the scan shown in Figure 2a of the main text. (a-c) show data directly presented in Figure 2a of the main text, included here for convenience. Included in this figure are (a) a scanning electron microscope (SEM) image of the scanned area (i.e., taken after the SECCM scan), showing droplet residues left behind by landings of the nanopipette; (b) an SECCM colour map showing current responses of each probe landing at HER potentials as individual pixels; (c) a comparative LSV showing the averaged HER responses of the grain boundary and two adjacent grains; and (d-f) individual voltammograms of the HER region for each of the three regions of interest, showing the average curves (black trace) that appear in (c) alongside the curves generated by plotting currents one standard deviation above and below the average (red dashed traces). All obtained electrochemical data were acquired using scan rate, υ = 1.2 V s^{-1}.

Section S3 – Discussion of the complexities in the determination of grain boundary geometry

Since grain boundaries are results of the intersection between two crystalline grains of a material in three-dimensional space, the complete structure of a grain boundary can only be defined by a total of five rotational degrees of freedom.[1,2] Three of these degrees of freedom define how the adjacent grains are oriented relative to each other, and the final two define the orientation of the intersection plane that makes up the grain boundary (though other interpretations of the degrees of freedom also exist). With such a variety of parameters affecting the overall geometry of each grain boundary, recognising trends in HER activity against grain boundary geometry becomes a problem of recognising trends in behaviour over five-dimensional space, if each degree of freedom is 'plotted' as its own axis in such a scenario.[3] Whilst this has been done in grain boundary studies in other fields (particularly notable examples being studies of the relationship between surface excess energy and the geometry of grain boundaries),[4,5] reliably identifying a structure-activity relationship for electrochemical properties would require an extremely large set of data.[6]

Further complicating the search for a grain boundary structure-activity relationship is the difficulty in measuring the five degrees of freedom of grain boundary geometry. *Electron backscatter diffraction* (EBSD) is available as a technique for measuring the three-dimensional orientations of crystalline grains, allowing for three of the aforementioned degrees of freedom to be conveniently found.[7] A fourth degree of freedom can be obtained through SEM or EBSD imaging of a grain boundary and noting the direction that the defect is oriented on the surface. However, the final degree of freedom is only found through observing the direction that the boundary extends internally in the material, and thus requires techniques such as *focused ion beam* (FIB) milling in order to expose and measure internal structures.[8] This presents an extremely labour-intensive endeavour since this needs to be performed separately on each boundary of interest. Foregoing FIB milling and accepting a slightly lower quantity of information on grain boundary geometry is possible, though is limited in the information that can be determined in structure-activity relationships.[9] Figure S3-1 graphically presents the limitations associated with relying solely on EBSD imagery for grain boundary analysis.

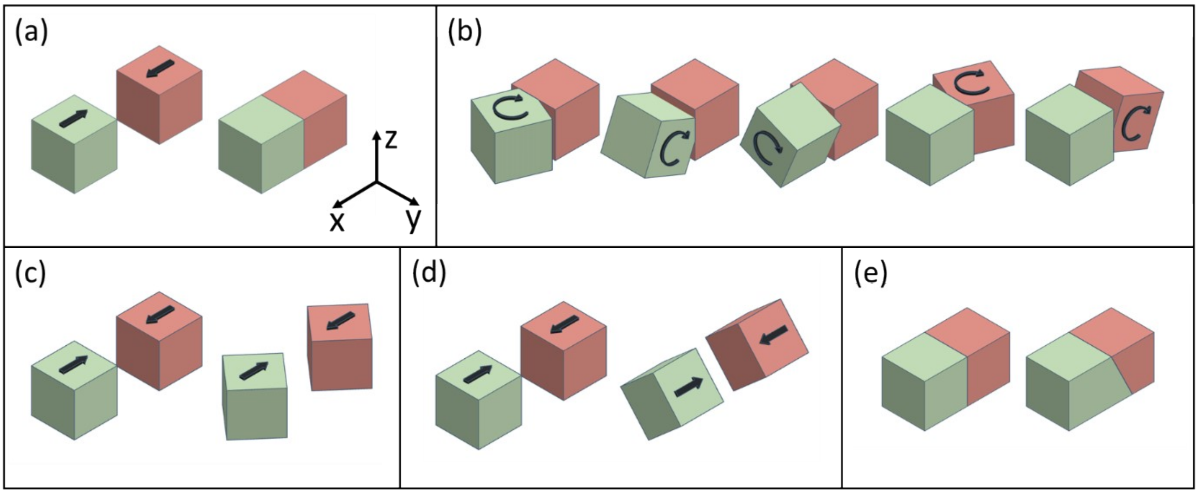

Figure S3-1: (a) Simple geometric depiction of a grain boundary, in which two perfect cubic crystals (green and red cubes) are forced to intersect on a planar boundary. (b) One possible depiction of the five degrees of rotational freedom exhibited by a grain boundary. In cases where the exact geometry of a grain boundary is required, all five rotation components must be exactly defined. (c) Simple example of the construction of two grain boundaries that an EBSD IPF map would be incapable of determining the difference between. Despite intersecting at different angles, all four crystallites have the same top-down orientation, and would be indiscernible in an IPF map. (d) Simple example of two identical grain boundaries that would appear different on an EBSD IPF map, due to being viewed at different orientations. (e) An example of two grain

boundaries that are completely indiscernible by any EBSD technique, since the underlying geometry is inaccessible from surface-confined analysis.

Despite the aforementioned limitations of performing structural analysis of grain boundaries solely by EBSD, FIB milling was not used in this investigation. The study opted to sacrifice a full geometric description of grain boundaries in favour of searching for weak correlations and trends that could be found in the limited information provided by EBSD alone. Ultimately, this decision proved inconsequential for the discussed results, though future studies on grain boundary electrochemistry may wish to employ a more complete technique. In fact, the combination of EBSD and FIB milling has been used in past studies on grain boundary electrochemistry, and represents a model procedure for this type of analysis.[10]

Section S4 – A macroscopic comparison of surface preparation techniques used in this study

Data obtained and presented in the main text utilise two different preparation procedures for the platinum surface being studied. The first involves the flame annealing (using a butane flame) of the platinum before immediately quenching the material in ultrapure water, whilst the second (designed to minimise the ridge-like topography of grain boundaries that occurs upon very fast cooling of the material) avoids the quenching step. It is suggested that whilst the composition and distribution of grains and grain boundaries should be different between these two preparation methods, it is assumed that the difference does not meaningfully impact the observed electrochemical properties of the surface on the macroscale. To demonstrate this, a platinum wire working electrode was subjected to both preparation techniques and cycled through its electrochemical window in sulfuric acid in each case. A comparison of the obtained voltammograms is provided in Figure S4-1.

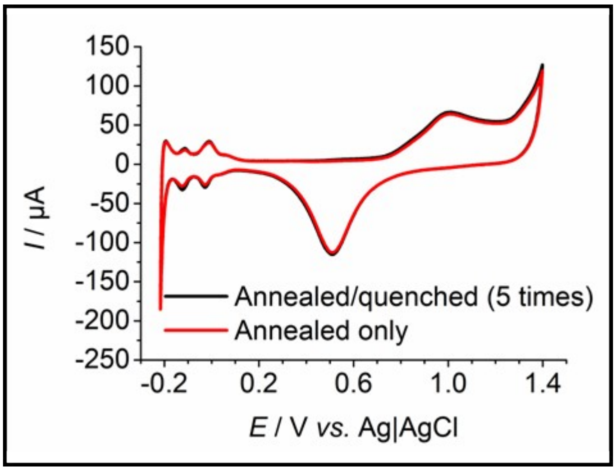

Figure S4-1: Comparative voltammograms obtained using a platinum wire working electrode (10 mm length, 0.5 mm width) in 0.5 M sulfuric acid. The comparison provides voltammograms generated from the 10th cycle of the entire electrochemical window under these conditions, obtained using a voltammetric scan rate of 0.25 V s^{-1}. The two examples show the result of the working electrode being repeatedly flame annealed and immediately quenched in deionised water a total of five times (black), and the case where a newly prepared wire was annealed only once without the quenching step (red).

As observed in Figure S4-1, the responses obtained for all processes within the electrochemical window of the platinum working electrode in sulfuric acid electrolyte are extremely consistent. Very little change is observed between the voltammograms obtained from each preparation technique.

Section S5 – Simulation of electrochemical surface cleaning in SECCM scans of grain boundaries using multiple voltammetric cycles

Whilst all SECCM scans shown in the main text are presented using data from the immediate cathodic sweep applied to the working electrode upon probe contact (i.e., they relate to a platinum surface that has not undergone any formation of an oxide layer), a question may arise over whether the trends in grain boundary activity change upon electrochemical cycling of the platinum surface. By cycling into potential regions that form (and then subsequently destroy) an oxide layer, atomic scale changes in structure occur, alongside physical cleaning of the surface, that may influence the conclusions drawn from this investigation.

Herein, small-scale simulation of electrochemical surface cleaning was undertaken by performing multiple voltammetric cycles on each landing the scans. In this way, comparative HER activity can be examined on the first and second cycles of a given grain boundary scan to see if any differences arise between the 'fresh' platinum surface and the surface after cycling.

For convenience, this will be demonstrated using a scan that will be analysed extensively in later sections (Figures S7-1 and S7-2), also appearing in Figure 5c of the main text. In section S7, the first cycle of the scan is examined, and a simplified analysis of this is shown in Figure S5-1. In particular, Figure S5-1d is most important for discussion, as it shows the average grain boundary HER response lying somewhere between the average HER responses of the two surrounding grains.

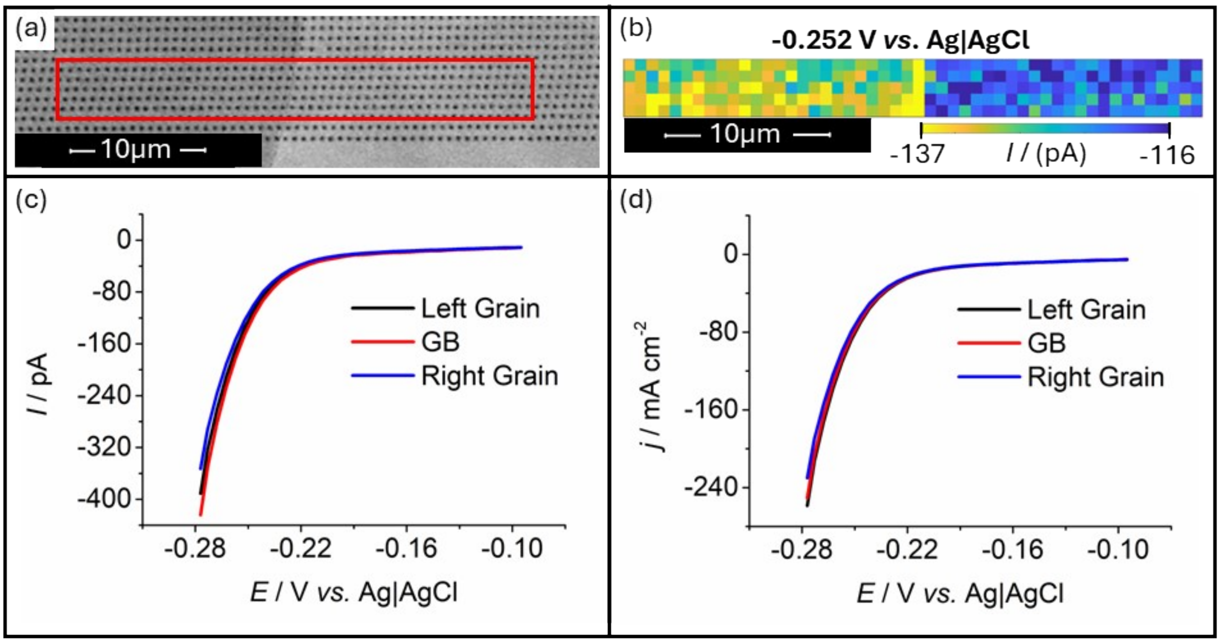

Figure S5-1: Extended analysis of an SECCM scan section also shown in Figures S7-1 and S7-2, alongside Figure 5c of the main text, noting that all data refer to the first cycle of the voltammograms undertaken. Images show (a) an SEM image of the analysed scan section; (b) an SECCM colour map of the GB-including scan region, showing current responses of each probe landing at HER potentials (-0.252 V vs Ag|AgCl) as individual pixels; (c) a comparative LSV showing the averaged HER responses of the grain boundary and two adjacent grains, showing the current obtained on the first cycle cathodic sweep; and (d) a copy of the voltammograms shown in (c), expressed in terms of current density instead of raw current. All obtained electrochemical data were acquired using scan rate, $\upsilon = 10$ V s^{-1}.

Since the scan shown in Figure S5-1 included a second voltammetric cycle taken on each individual landing, voltammograms can also be presented that show the HER behaviour on the second cycle. This is shown in Figure S5-2.

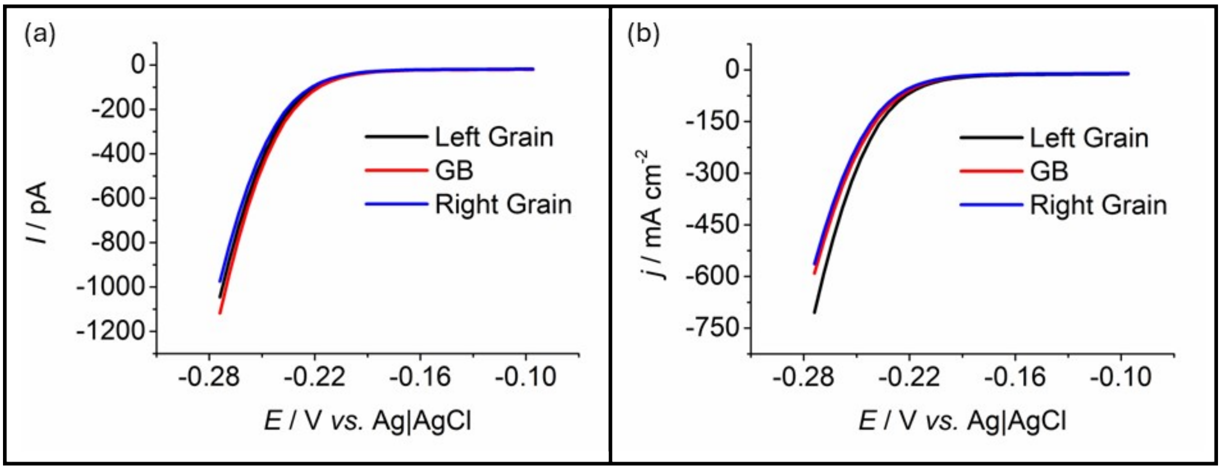

Figure S5-2: Voltammograms identical to those shown in Figures S5-1c and S5-1d, wherein the data presented instead relates to the second voltammetric cycle. Images show (a) a comparative LSV showing the averaged HER responses of the grain boundary and two adjacent grains, showing the current obtained on the second cycle cathodic sweep; and (b) a copy of the voltammograms shown in (a), expressed in terms of current density instead of raw current. All obtained electrochemical data were acquired using scan rate, υ = 10 V s^{-1}.

Through the analysis of Figure S5-2, it is observed that the same conclusions can be drawn from the second voltammetric cycle as those obtained from the first. In both cases, the apparent small degree of increased activity seen on the grain boundary measurements in Figures S5-1c and S5-2a is no longer apparent when accounting for electrochemical surface area, as seen in Figures S5-1d and S5-2b respectively. Aligning with the conclusions made in the extended analysis of the first cycle in Section S7, the second cycle also shows that the current density response on this boundary appears to be between the two adjacent grains, as one may expect from a boundary that does not yield enhanced activity on this scale.

Section S6 – Extended data analysis on scans shown in Figure 6

Figure 6 in the main text provides a simplified overview of thirteen scans that each included a grain boundary. More thorough analysis of each scan is given in the following six supporting figures. These provide SEM imagery, SECCM activity maps, and appropriate LSVs of every line graph presented in Figure 6. All examples are given in the order that they appear (from top to bottom) in Figure 6.

A total of 5 SECCM probes (denoted probes 1-5) were used for the acquisition of these 12 scans, each with an approximate tip diameter of 250 nm. For each scan in the following, the probe is specified.

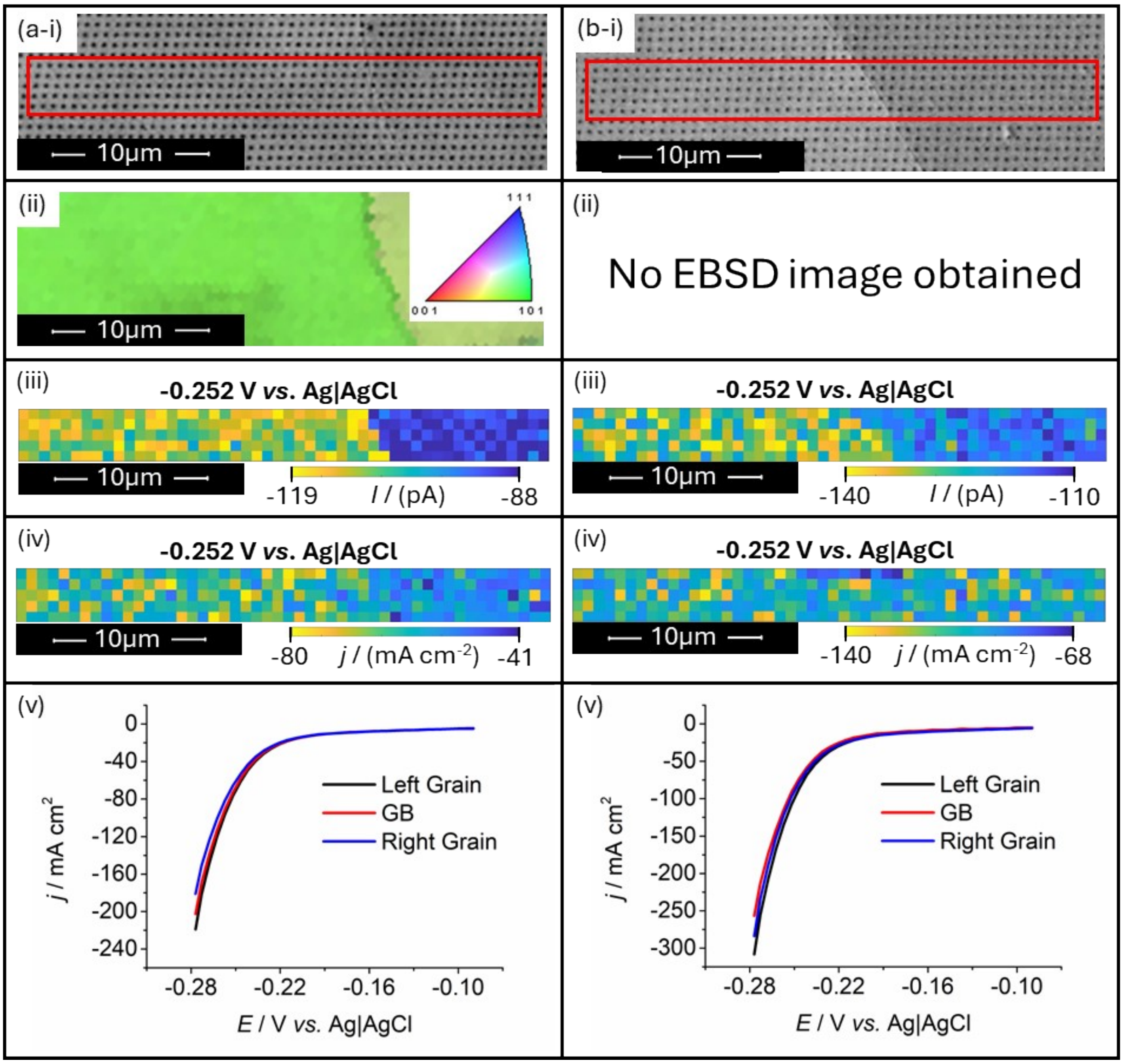

Figure S6-1: Extended analysis of two grain boundaries shown in Figure 6—(a) and (b) respectively. Scan (a) was taken using probe 1, whilst scan (b) was taken using probe 2. In each case, images show (i) an SEM image with the analysed region of the SECCM scan highlighted; (ii) a co-located EBSD image of the scanned area to provide conclusive indication of the location of the grain boundary within the scan; (iii) an SECCM colour map of the GB-including scan region, showing current responses of each probe landing at HER potentials (-0.252 V vs Ag|AgCl) as individual pixels; (iv) an alteration of the SECCM colour map made by applying surface area correction described in the main text, plotting each pixel with colours relating to current density instead of current alone; and (v) a comparative LSV showing the averaged HER responses of the grain boundary and two adjacent grains.

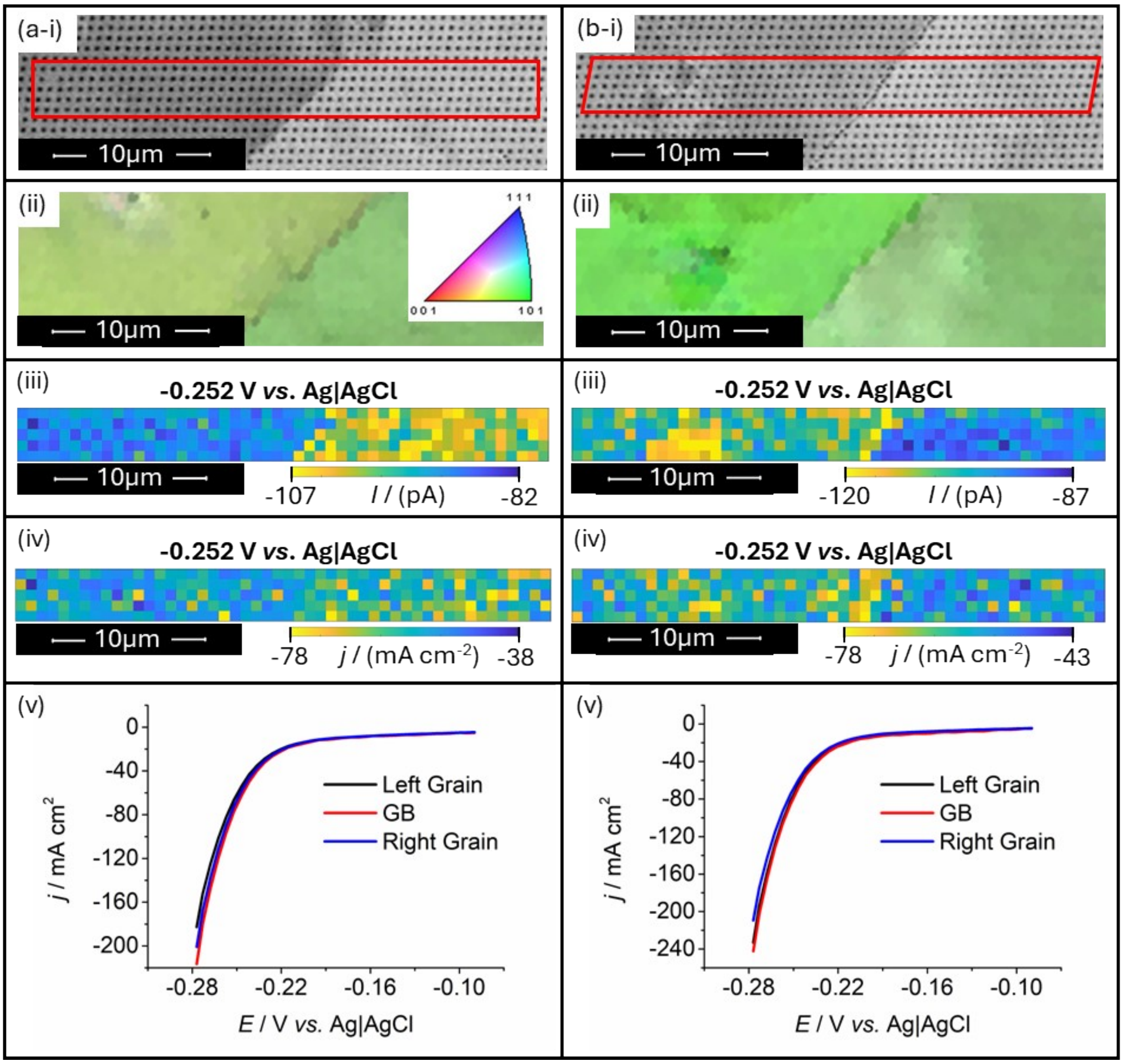

Figure S6-2: Extended analysis of two grain boundaries shown in Figure 6—(a) and (b) respectively. Both scans were taken using probe 1. In each case, images show (i) an SEM image with the analysed region of the SECCM scan highlighted; (ii) a co-located EBSD image of the scanned area to provide conclusive indication of the location of the grain boundary within the scan; (iii) an SECCM colour map of the GB-including scan region, showing current responses of each probe landing at HER potentials (-0.252 V vs Ag|AgCl) as individual pixels; (iv) an alteration of the SECCM colour map made by applying surface area correction described in the main text, plotting each pixel with colours relating to current density instead of current alone; and (v) a comparative LSV showing the averaged HER responses of the grain boundary and two adjacent grains.

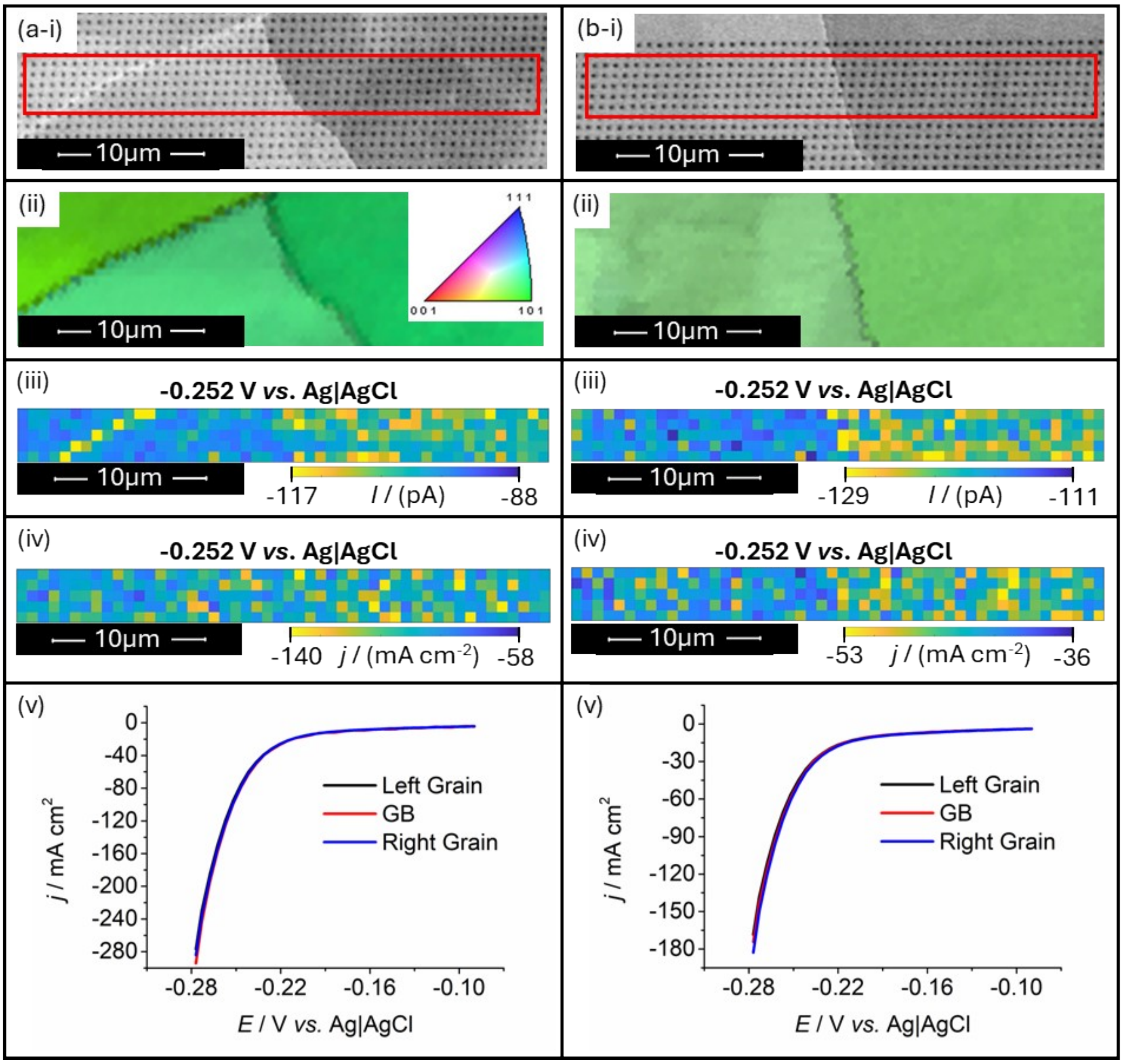

Figure S6-3: Extended analysis of two grain boundaries shown in Figure 6—(a) and (b) respectively. Scan (a) was taken using probe 3, whilst scan (b) was taken using probe 4. In each case, images show (i) an SEM image with the analysed region of the SECCM scan highlighted; (ii) a co-located EBSD image of the scanned area to provide conclusive indication of the location of the grain boundary within the scan; (iii) an SECCM colour map of the GB-including scan region, showing current responses of each probe landing at HER potentials (-0.252 V vs Ag|AgCl) as individual pixels; (iv) an alteration of the SECCM colour map made by applying surface area correction described in the main text, plotting each pixel with colours relating to current density instead of current alone; and (v) a comparative LSV showing the averaged HER responses of the grain boundary and two adjacent grains.

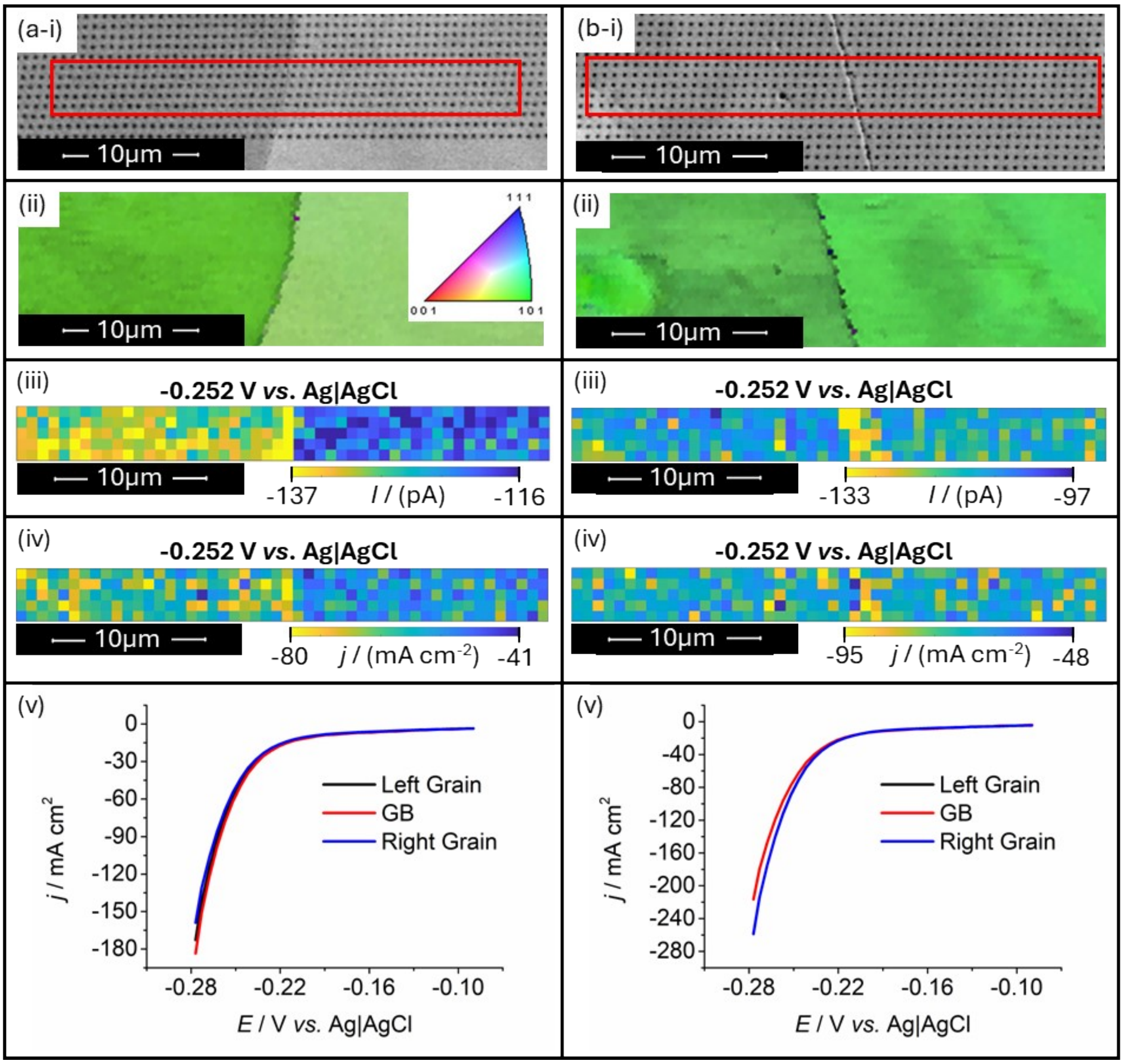

Figure S6-4: Extended analysis of two grain boundaries shown in Figure 6—(a) and (b) respectively. Scan (a) was taken using probe 4, whilst scan (b) was taken using probe 1. In each case, images show (i) an SEM image with the analysed region of the SECCM scan highlighted; (ii) a co-located EBSD image of the scanned area to provide conclusive indication of the location of the grain boundary within the scan; (iii) an SECCM colour map of the GB-including scan region, showing current responses of each probe landing at HER potentials (-0.252 V vs Ag|AgCl) as individual pixels; (iv) an alteration of the SECCM colour map made by applying surface area correction described in the main text, plotting each pixel with colours relating to current density instead of current alone; and (v) a comparative LSV showing the averaged HER responses of the grain boundary and two adjacent grains.

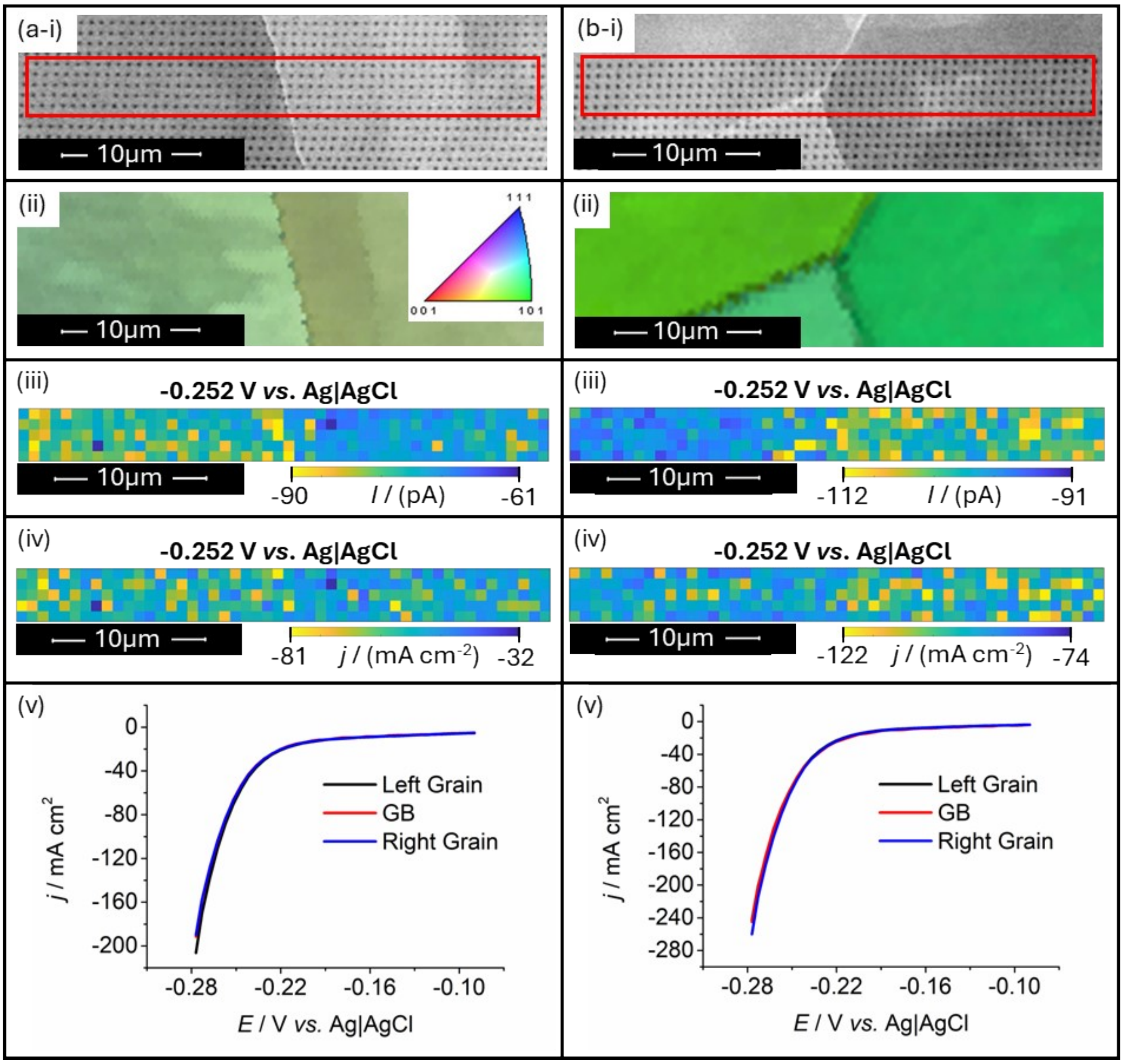

Figure S6-5: Extended analysis of two grain boundaries shown in Figure 6—(a) and (b) respectively. Scan (a) was taken using probe 5, whilst scan (b) was taken using probe 3. In each case, images show (i) an SEM image with the analysed region of the SECCM scan highlighted; (ii) a co-located EBSD image of the scanned area to provide conclusive indication of the location of the grain boundary within the scan; (iii) an SECCM colour map of the GB-including scan region, showing current responses of each probe landing at HER potentials (-0.252 V vs Ag|AgCl) as individual pixels; (iv) an alteration of the SECCM colour map made by applying surface area correction described in the main text, plotting each pixel with colours relating to current density instead of current alone; and (v) a comparative LSV showing the averaged HER responses of the grain boundary and two adjacent grains.

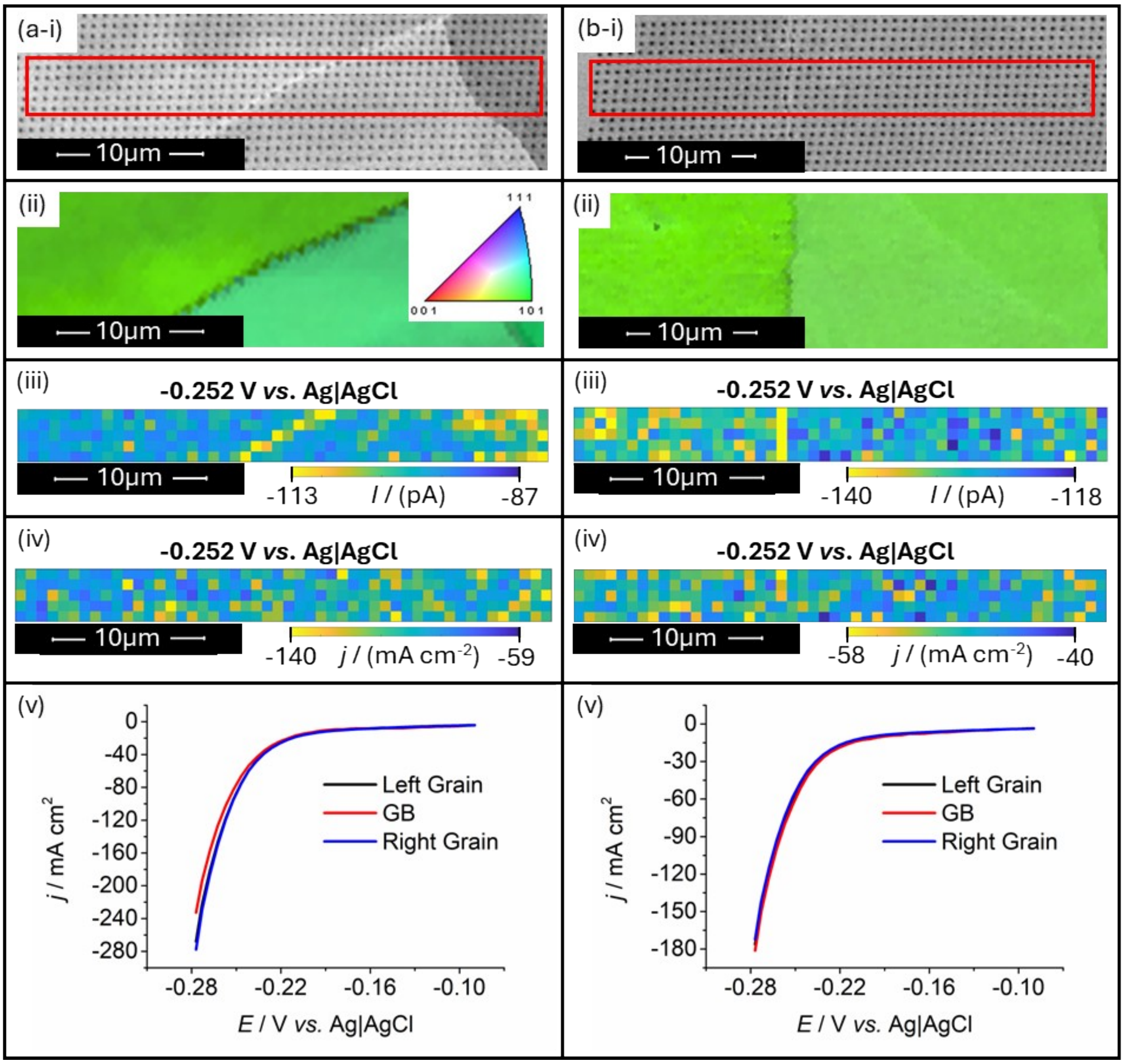

Figure S6-6: Extended analysis of two grain boundaries shown in Figure 6—(a) and (b) respectively. Scan (a) was taken using probe 3, whilst scan (b) was taken using probe 4. In each case, images show (i) an SEM image with the analysed region of the SECCM scan highlighted; (ii) a co-located EBSD image of the scanned area to provide conclusive indication of the location of the grain boundary within the scan; (iii) an SECCM colour map of the GB-including scan region, showing current responses of each probe landing at HER potentials (-0.252 V vs Ag|AgCl) as individual pixels; (iv) an alteration of the SECCM colour map made by applying surface area correction described in the main text, plotting each pixel with colours relating to current density instead of current alone; and (v) a comparative LSV showing the averaged HER responses of the grain boundary and two adjacent grains.

Section S7 – Demonstration of the calculation of C_{DL} and its use in scaling data to the electrochemical surface area of contact

The full procedure for the calculation of double layer capacitance (C_{DL}) and using it for scaling HER activity data is presented as follows for two SECCM scan sections shown in the main text. Note that in these examples, averaged voltammograms of the three regions of interest (the grain boundary and the two surrounding grains) are analysed and processed, whereas in the main text and sections S1 and S6, this analysis and processing is done on a landing-by-landing basis. Analysis of averages is simply performed here for convenience of demonstration, since presenting and processing every individual CV in a given scan would be impractical to show visually.

First, the scan section processed and shown in the second section of Figure 5 in the main text (with mapped data directly presented in Figures 5c and 5e) is used as an example.

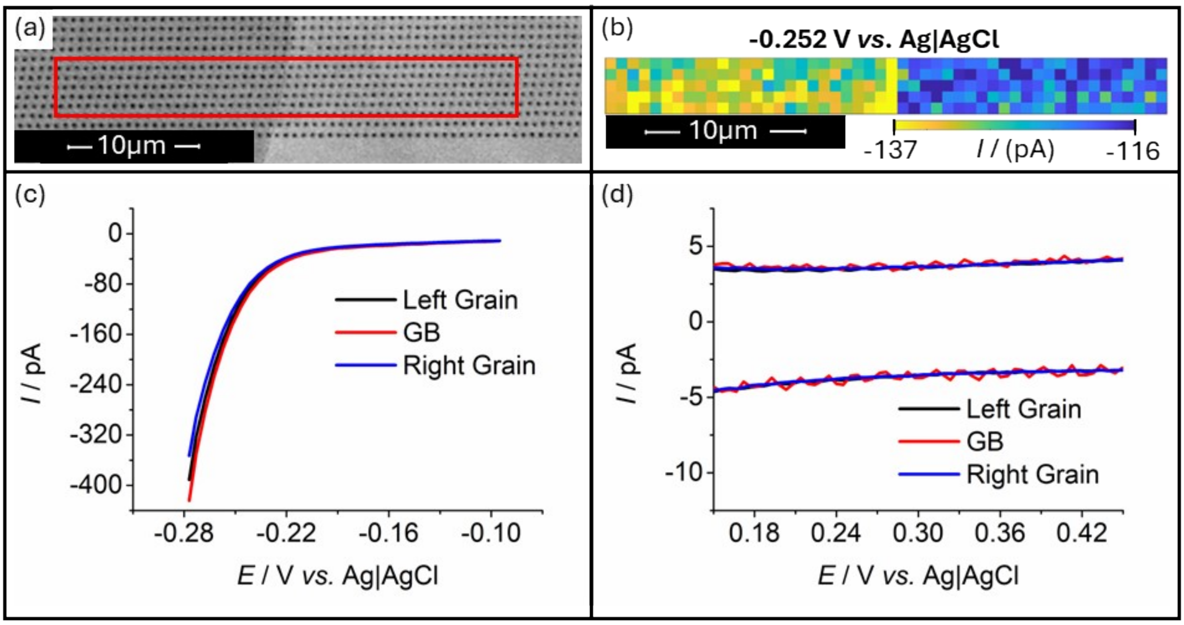

Figure S7-1: Extended analysis of an SECCM scan section shown in Figure 5c of the main text. Images show (a) an SEM image of the analysed scan section; (b) an SECCM colour map of the GB-including scan region, showing current responses of probe landings at HER potentials (-0.252 V vs Ag|AgCl) as individual pixels; (c) a comparative LSV showing the averaged HER responses of the grain boundary and two adjacent grains, showing the current obtained on the cathodic sweep; and (d) a comparative CV zoomed in on the double layer charging region described in the main text (0.15-0.45 V vs Ag|AgCl), showing the current obtained on both the cathodic (lower) and anodic (upper) sweeps. All obtained electrochemical data were acquired using scan rate, $\upsilon = 10$ V s^{-1}.

Taking average current magnitudes ($|i_{DL}|$) from the double layer charging region in Figure S7-1d (from both the cathodic and anodic sweep) allows for the use of Equation S7-1 to estimate the average C_{DL} exhibited by the landings within each of the three regions of the shown scan. Note that stray capacitance (i.e., the capacitance exhibited by the parts of the system not within the electrochemical cell, C_{stray}) must be subtracted from the overall capacitance observed (C_{total}) in order to get a true reading of C_{DL}. In this case, C_{stray} was measured by performing voltammetric cycling when the SECCM probe was not in contact with the surface, yielding a value of $C_{stray} = 0.3$ pF.

With C_{DL} found, Equation S7-2 (a modified form of Equation 1 in the main text) can be used to estimate the average surface area of droplet contact. The specific double layer capacitance (C_A) of the platinum surface is taken to be 44 μF cm^{-2}, as described in the main text.

$$C_{DL} = C_{total} - C_{stray} = \frac{|i_{DL}|}{\upsilon} - C_{stray} \tag{S7-1}$$

$$A = \frac{C_{DL}}{C_A} \tag{S7-2}$$

By these definitions, the average C_{DL} values for each region (left grain, GB, right grain) are then found and presented in Table S7-1, alongside the estimated areas of droplet contact.

Table S7-1: Estimation of various parameters of the three regions of interest (left grain, GB, and right grain) within the scan shown in Figure S7-1.

	Left Grain	GB	Right Grain
$\lvert i_{DL}\rvert$ (pA)	3.66	3.74	3.67
C_{DL} (pF)	0.066	0.074	0.067
Estimated area of contact (µm^2)	0.150	0.168	0.152
Estimated diameter of contact (nm)	437	462	440

Using the estimated areas of droplet contact found in Table S7-1, one can now adjust the voltammograms shown in Figure S7-1c to yield voltammograms showing current density on the y axis. This is performed through the use of Equation 2 from the main text. These adjusted voltammograms are provided in Figure S7-2.

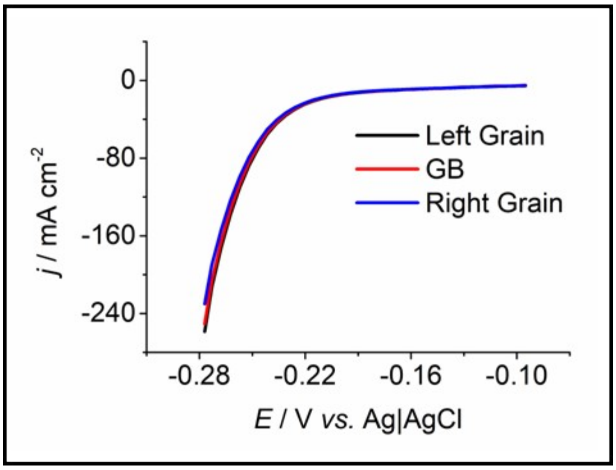

Figure S7-2: Adjusted LSVs of data presented in Figure S7-1c, presenting current density on the y axis instead of raw current, calculated via the estimated areas of contact shown in Table S7-1.

From Figure S7-2, it is observed that the apparent small degree of increased activity seen on the grain boundary measurements in Figure S7-1c is no longer apparent when accounting for electrochemical surface area in this way. Instead, the current density response on this grain boundary appears to be a weighted average of the two adjacent grains, as one may expect from a boundary that does not yield enhanced activity on this scale.

As alluded to in the main text, this method of surface area compensation fundamentally relies on the assumption that the current magnitude associated with the formation of the electrochemical double layer is dependent on surface area alone (at a given voltammetric scan rate). However, this assumption is known not to hold perfectly for all cases, since this quantity can vary with different surface structures (i.e. different grain orientations and content of defects). Nonetheless, on well-defined SECCM landings of similar contact area, the relative differences in double layer capacitance are observationally much smaller than differences in HER activity, and the two quantities are not seen to be directly correlated. Hence, even though some error may be introduced by using this assumption, the overall trends observed across the multitude of grain boundaries analysed in this study are unlikely to be significantly impacted.

Since the presented scan did not exhibit much variation in crystallographic orientation (refer to the EBSD image provided in Figure 5a-ii in the main text), the two grains did not show much variation in HER activity. In fact, this is true for all cases shown in Figures 5 and 6 of the main text, as most grains were oriented close to the 101 orientation on the surface. Additionally, this scan section did not suggest a particularly high degree of apparent enhanced grain boundary activity before surface area correction techniques were performed. Given this, it is also deemed prudent to provide an example of a scan in which two grains are included with significantly different surface orientations (and hence, HER activities), along with an apparent enhancement in activity on the grain boundary, to show that the method of using C_{DL} as a measure of surface area does not simply cancel out differences in HER activity.

The second scan section selected to demonstrate this can be found in Figure 3b of the main text. Once again, the data obtained from this region is shown in Figure S7-3. Note that only the anodic sweep of the double layer charging region (0.15-0.45 V vs. Ag|AgCl) was selected for analysis in this scan due to the fact that some minor faradaic contribution from the reduction of platinum oxide was observed on the cathodic sweep for some landings.

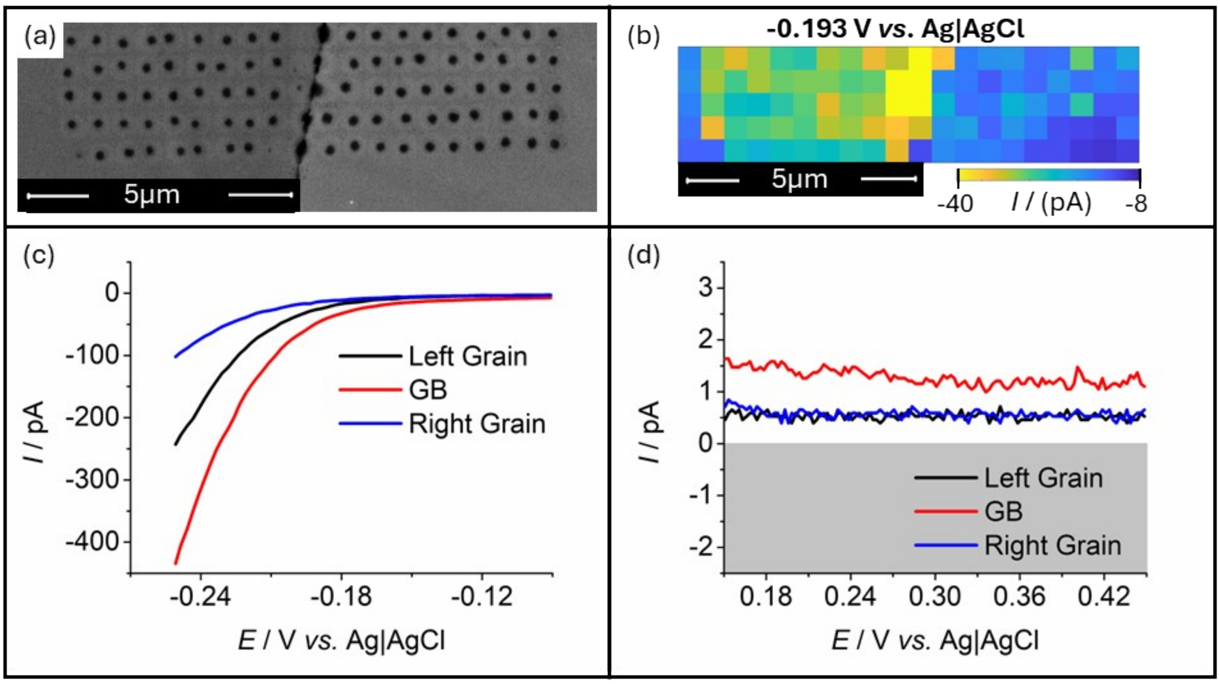

Figure S7-3: Extended analysis of an SECCM scan section shown in Figure 3b of the main text. Images show (a) an SEM image of the analysed scan section; (b) an SECCM colour map of the GB-including scan region, showing current responses of probe landings at HER potentials (-0.193 V vs Ag|AgCl) as individual pixels; (c) a comparative LSV showing the averaged HER responses of the grain boundary and two adjacent grains, showing the current obtained on the cathodic sweep; and (d) a comparative voltammogram zoomed in on the double layer charging region described in the main text (0.15-0.45 V vs Ag|AgCl), showing the current obtained on the anodic sweep. All obtained electrochemical data were acquired using scan rate, υ = 1.2 V s^{-1}.

Using Equations S7-1 and S7-2, the average C_{DL} values for each region (left grain, GB, right grain) are then found and presented in Table S7-2, alongside the estimated areas of droplet contact.

Table S7-2: Estimation of various parameters of the three regions of interest (left grain, GB, and right grain) within the scan shown in Figure S7-3.

	Left Grain	GB	Right Grain
$\|i_{DL}\|$ (pA)	0.52	1.28	0.57
C_{DL} (pF)	0.141	0.768	0.175
Estimated area of contact (μm^2)	0.321	1.74	0.400
Estimated diameter of contact (nm)	639	1490	713

It may be noted here that the droplet contact areas are found to be larger than those found in the previous analysis (i.e., the data shown in Table S7-1). This is likely a result of the different methods of surface preparation, since the platinum surface referred to in Table S7-2 was prepared by annealing and quenching in water, and the surface referred to in Table S7-1 skipped the quenching step (as described in the Experimental section). It is likely that the quenching step led to an increase in the electrochemical roughness factor.

Finally, voltammograms from Figure S7-3c are adjusted to yield corrected voltammograms through the estimated areas of contact in Table S7-2 and Equation 2 from the main text. These adjusted voltammograms are provided in Figure S7-4.

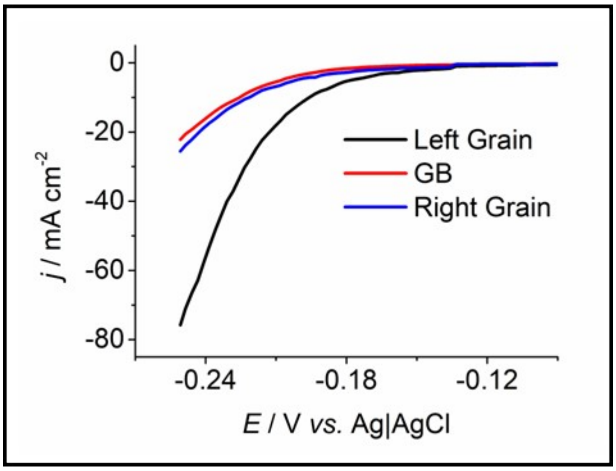

Figure S7-4: Adjusted LSVs of data presented in Figure S7-3c, presenting current density on the y axis instead of raw current, calculated via the estimated areas of contact shown in Table S7-2.

Clearly shown here is the fact that differences in HER activity between grains (i.e., left grain and right grain in Figures S7-3 and S7-4) remain obvious after applying this type of correction. In fact, this case shows an example where the intergranular difference becomes more pronounced after correcting for electrochemical surface area in this way. Hence, any argument one might make about the fact that HER activity may be correlated with C_{DL} (rendering this technique merely a way of cancelling out differences in HER activity) cannot be universally true. Due to this, it is suggested that the use of C_{DL} as a means of correcting for surface area is a valid technique that does not detrimentally skew data away from observing spatial differences in activity.

The grain boundary response in Figure S7-4 appears to indicate a similar activity to that observed on the right grain, despite it generally being expected that an 'inactive' boundary would yield something close to the average of the two adjacent grains. This may indicate that the wetting of the grain boundary landings within this scan approach the limit of what this surface area correction technique can reliably account for. As such, avenues for keeping wetting minimal were explored as described in the main text.

Section S8 – Attempts at using alternative techniques for scaling data to the electrochemical surface area of contact

Whilst the use of the measured double layer capacitance was deemed the most appropriate means of estimating the electrochemical surface area of individual droplet landings, other methods of this estimation do exist, and may be more convenient under some circumstances. Namely, one could estimate surface areas of contact by analysing the sizes of droplet residues imaged via SEM (as noted in the main text), or a more conventional route may be to perform this estimation by inference from the charge transferred in hydrogen underpotential deposition (H_{UPD}) surface processes. These techniques were avoided due to fundamental issues with their practical implementation when working on this scale of SECCM. A full discussion of these issues, along with a comparison of these techniques to the employed method using double layer capacitance, are provided for both alternative methods as follows.

Estimation of surface area of electrochemical cell contact via SEM imagery

First, the estimation of contacted surface area by SEM imaging of landing residues is described as an ideal method in the main text, though it is noted as being too cumbersome to perform on every single landing. Other issues with this method exist, however, and the pitfalls are best demonstrated by attempting to analyse a grain boundary scan from the previous section.

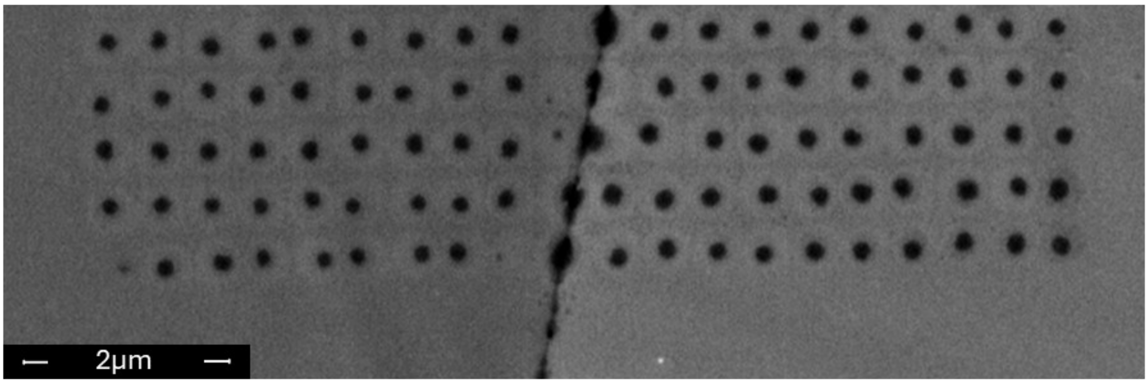

Figure S8-1: Enlarged view of the SEM image depicting the SECCM scan area analysed in Figure S7-3 and Figure 3b of the main text.

In the enlarged view of the second scan shown in Section S7, shown in Figure S8-1, the locations of droplet residues (left behind after the SECCM probe has landed, performed voltammetric cycling, and then lifted again) are clear, indicating the exact locations of probe landings. Whilst it may be easy to assume that the dark spots are representative of the areas contacted by the relevant droplet during measurement, this is actually what remains of the droplet after evaporation of the electrolyte's water content. Instead, a more accurate representation of the contacted area is the generally circular 'halo' or 'shadow' around each dark point. It is suggested that these somewhat larger regions represent the area of the surface that has been altered or cleaned by electrochemical cycling within the droplet, and these regions are visibly quite well defined on the areas within each grain. For the landings within each grain, it is reasonable enough to take a few points and analyse the contacted surface area that the image suggests. This is performed in Figure S8-2, wherein these estimated areas are also compared to the areas obtained from double layer capacitance-based measurements in Table S7-2.

(a)

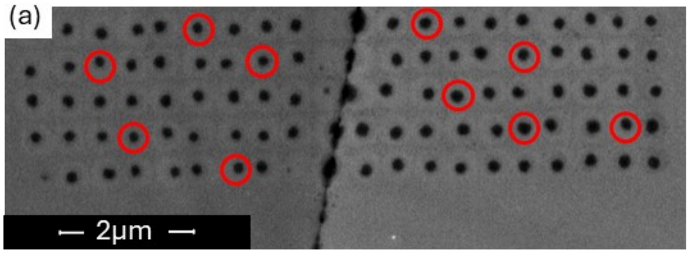

(b)

	Left Grain	Right Grain
Image area (μm^2)	0.21	0.22
C_{DL}-estimated area (μm^2)	0.321	0.400

Figure S8-2: Estimation of the contacted areas of a selection of probe landings. (a) presents the image shown in Figure S8-1, highlighting the points selected for analysis (5 points for each grain), while (b) tabulates the average areas of these landings suggested by the image, comparing to the area values found in Table S7-2.

Performing an image-based area estimation for the selection of landings shown in Figure S8-2 is straightforward due to the fact that the areas of contact are relatively well-defined. Interestingly, the areas estimated by the method involving double layer capacitance are consistently higher than the areas that the image would suggest (as observed in Figure S8-2b). This discrepancy is likely a result of a combination of nanoscale roughness and an imperfect estimation of the specific capacitance of the platinum surface, currently assumed to be 44 μF cm^{-2}.

This method of area estimation is effective, though tedious, for SECCM probe landings that occur within a grain. However, the same cannot be said for landings that include a grain boundary. As observed in Figure S8-2a, the residues left behind by probe landings on a grain boundary are not clearly defined in terms of size or shape. In some cases, the previously mentioned 'shadow' is roughly observable, though the extent of spreading along a boundary is largely unclear, as is the actual geometric profile of the boundary. Additionally, it is completely unknown whether the apparent area of contact is a direct representation of the contact that occurred during measurement, or if some 'spreading' of electrolyte occurred in the time between taking the measurement and performing SEM imagery.

An enlarged view of the boundary landings shown in Figure S8-2a is provided in Figure S8-3a, wherein (i) and (ii) denote examples of boundary landings where the areas of contact cannot be easily estimated visually. In example (i), the extent of wetting along the boundary is completely unclear and there is even evidence of some electrolyte wetting to the left of the image far beyond the regular droplet diameter seen in other well-defined landings. In example (ii), some indication of a circular 'shadow' is visible, though it is obvious that much of the electrolyte has spread to and along the boundary line, the exact extent of this being unclear.

Figure S8-3 also includes two other examples of scan sections that include a grain boundary (Figures S8-3b and S8-3c), with their own highlighted instances. The landings in Figure S8-3b are much larger than in scans previously discussed, made using a probe tip of approximately 1000 nm diameter. Landing examples (iii) and (iv) are actually well-defined despite including a boundary within, showing that this technique of area estimation by SEM may be applicable to larger probes. However, as discussed in the main text, larger probes (and the resulting larger areas of contact) minimise the ability to observe enhanced activity of grain boundaries, since the grain boundaries themselves take up a much smaller fraction of the contacted surface.

Finally, Figure S8-3c is an example of another scan in a very similar format to the one shown in Figure S8-3a. Landing example (v) is once again very poorly defined due to electrolyte wetting into the boundary. Accurate estimation of surface area of contact is deemed impossible here.

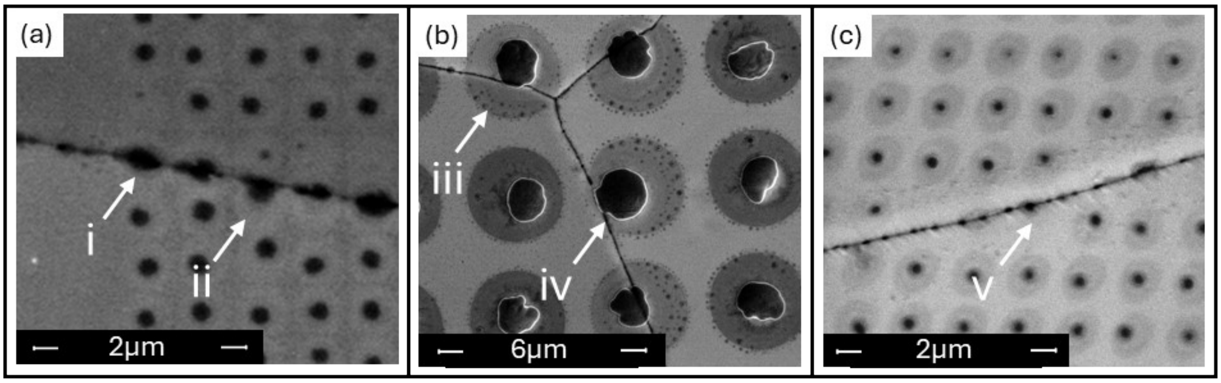

Figure S8-3: Three examples of SEM images (a, b, and c) containing enlarged views of SECCM scan areas that contain grain boundaries.

Overall, whilst estimation of contacted areas of probe landings by SEM imagery may have some utility in areas that do not contain grain boundaries, and may even be applicable to areas that do contain grain boundaries when working with larger probes (tip diameter ≥ 1 µm), it is impractical for use in scans that wish to study grain boundaries with nanoscale probe sizes. Since this study aimed to study grain boundaries via SECCM using the smallest probe diameters available (~200 nm), it was quickly apparent that this method of surface area correction was unviable.

Estimation of surface area of electrochemical cell contact via H_{UPD} peak integration

Macroscopically, the H_{UPD} processes, referring to the surface processes involving the adsorption and desorption of hydrogen atoms onto the working electrode surface at potentials more positive than the equilibrium potential of the HER, consist of well-defined peaks when performing voltammetric cycling in a sulfuric acid solution. Due to the fact that these are surface confined processes, integrating these peaks to obtain the total charge transferred for these processes is expected to yield a value proportional to the total area of the working electrode. As such, it stands to reason that this may also provide a convenient means of estimating the area of contact of SECCM probe landings. An attempt at performing surface area estimations in this manner is presented as follows.

For convenience, the selected SECCM scan section for this analysis is chosen to match the section that has already been analysed above through the use of double layer capacitance and SEM imagery (i.e., it is the same scan section as the example that appears in Figures S7-3. S8-1, and S8-2).

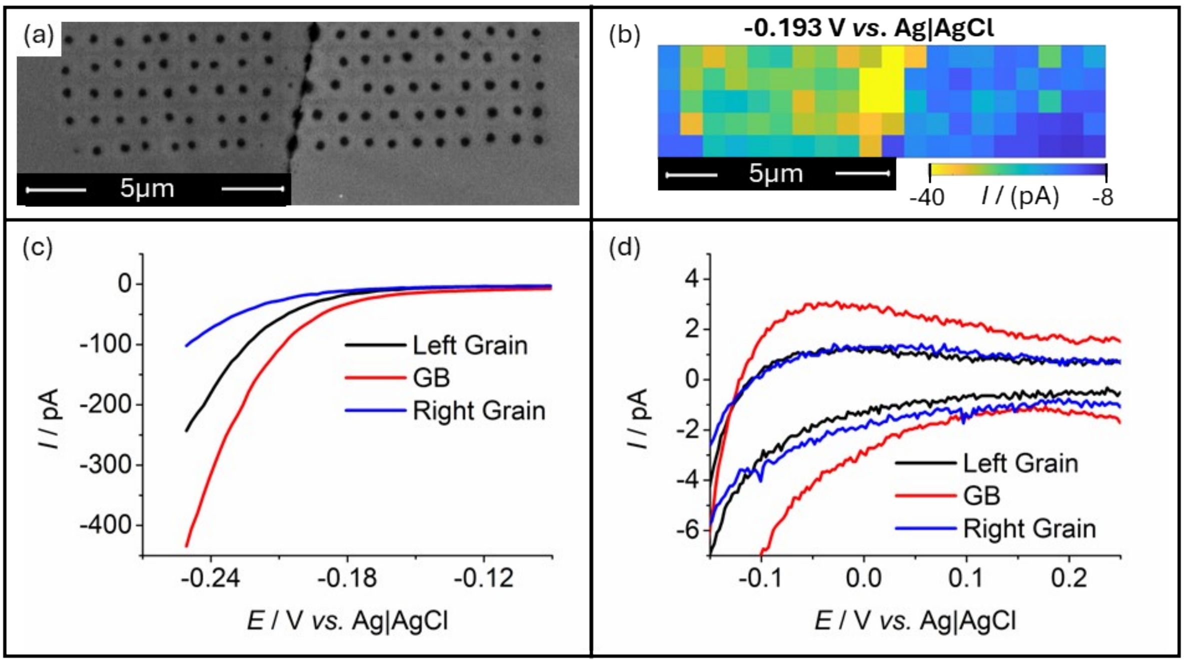

Figure S8-4: Extended analysis of an SECCM scan section shown in Figure 3b of the main text. Images show (a) an SEM image of the analysed scan section; (b) an SECCM colour map of the GB-including scan region, showing current responses of probe landings at HER potentials (-0.193 V vs Ag|AgCl) as individual pixels; (c) a comparative LSV showing the averaged HER responses of the grain boundary and two adjacent grains, showing the current obtained on the cathodic sweep; and (d) a comparative CV zoomed in on the H_{UPD} region (approx. -0.1 to +0.1 V vs Ag|AgCl), showing the current obtained on both the cathodic (lower) and anodic (upper) sweeps. All obtained electrochemical data were acquired using scan rate, υ = 1.2 V s^{-1}.

When looking at the H_{UPD} region (approx. -0.1 to +0.1 V vs Ag|AgCl) of the voltammograms shown in Figure S8-4d, an issue arises. When working on this scale and this scan rate, H_{UPD} peaks are very poorly defined. In terms of the cathodic sweep, it is unclear where the H_{UPD} region ends and the HER process begins, making integration of this area very difficult to do consistently. The H_{UPD} region is somewhat clearer on the anodic sweep though, showing a broad but obvious peak associated with the desorption of hydrogen atoms. However, even if one is to decide to integrate this anodic portion of the H_{UPD} region, another issue is clear in the fact that the current obtained in this region receives major contribution from non-faradaic (i.e., capacitive) processes. This contribution must be subtracted before integration is done, under the assumption that capacitive current is constant for all potentials.

Conveniently, the currents associated with capacitive charging (i_{DL}) have already been established for the three regions of interest in Section S7 (Table S7-2). By subtracting these as baseline currents and integrating the H_{UPD} peaks, figures for the charge passed in this region (Q_H) are shown in Table S8-1. Additionally, assuming that the formation/loss of a monolayer of hydrogen atoms is associated with the charge transfer of 210 µC cm^{-2} (generally accepted in literature as the conversion factor for polycrystalline platinum[11]), the estimated surface area of contact is also estimated and presented.

Table S8-1: Estimation of various parameters of the three regions of interest (left grain, GB, and right grain) within the scan shown in Figure S8-4.

	Left Grain	GB	Right Grain
Q_H (pC)	0.0541	0.1891	0.0647
H_{UPD}-estimated area of contact (µm^2)	0.0258	0.0901	0.0308
C_{DL}-estimated area of contact (µm^2)	0.321	1.746	0.400
SEM imaged area (µm^2)	0.21	Unclear	0.22

Under the assumptions made, the total charge transferred within the H_{UPD} (and the associated conversion to surface area) yields values for surface area of contact that are more than an order of magnitude lower than the estimates made by C_{DL} and SEM imagery. The estimations being significantly lower than the areas of the SEM-imaged residues is particularly worrisome, since this discrepancy cannot be readily rationalised by considering roughness factors. Due to this, the technique is considered unreliable when working on these scales.

Despite the clear disconnect between the surface area values obtained by H_{UPD} peak integration and the estimations established previously, one can still utilise these values in an attempt to correct for surface area in the same manner as was performed in Section S7 (i.e., voltammograms from Figure S8-4c can be expressed in terms of current density instead of current). This is depicted in Figure S8-5, alongside a comparison with the corrected voltammograms found previously through C_{DL}-corrections.

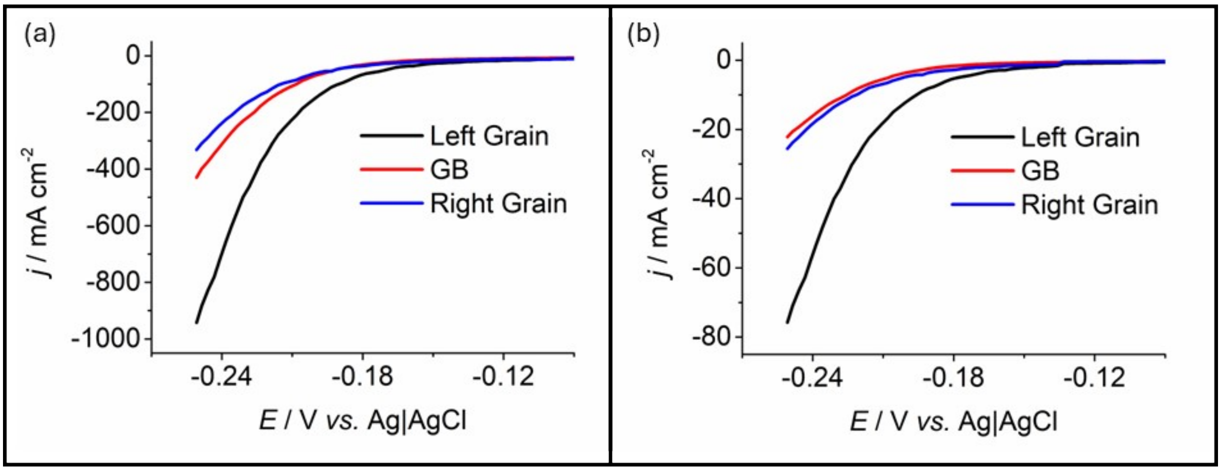

Figure S8-5: (a) Adjusted LSVs of data presented in Figure S8-4c, presenting current density on the y axis instead of raw current, calculated via the areas of contact derived from the H_{UPD} peaks (numerically presented in Table S8-1). (b) For comparative purposes, a copy of this data is provided in which the same current densities are measured via the C_{DL}-derived areas of contact (also numerically presented in Table S8-1).

Whilst the absolute values of current density obtained via H_{UPD} peak integration were found to be somewhat nonsensical (as observed on the scale of the y axis in Figure S8-5a), the relative difference between the activity of the left and right grain corresponds very closely with that obtained via C_{DL}-based estimates of surface area. Additionally, the activity of the grain boundary, in a relative sense, may also be considered to yield a more reasonable value using this method, since it now corresponds more closely with the weighted average activity of the two adjacent grains, as would be expected to occur for a boundary with no significantly enhanced activity on this scale. At the very least, both techniques certainly show agreement in showing that the grain boundary itself is not inherently more active than the surrounding grains.

In any case, the unreasonable current density scales obtained when utilising the integration of H_{UPD} peaks as a means of estimating surface area, alongside the complexity in its estimation on current scales this small (especially when one must consider the contribution of capacitive effects anyway), this technique was avoided in favour of C_{DL}-based area estimations in all examples discussed in the main text. Additionally, the example scan shown in S8-4 and S8-5 represents a scan section wherein voltammetric cycling was performed at 1.2 V s^{-1}, whereas the majority of scans in the main text utilised a voltammetric scan rate of 10 V s^{-1}, making the H_{UPD} peaks even more poorly defined. C_{DL}-based techniques were found to be much simpler to use, especially when excess wetting on grain boundaries was minimised.

Section S9 – Calculation of expected percentages of SECCM droplet areas taken up by a grain boundary

The foundational basis of SECCM's utility in measuring grain boundary activity largely comes from the fact that the area of the working electrode captured in the droplet cell of a single SECCM measurement is small enough such that a grain boundary can be considered to take up a meaningful fraction of the measured surface. With a droplet size defined by a diameter d, and a grain boundary width (or, the width of any electrochemically active region associated with a grain boundary) defined by w, the fraction of the measured area taken up by the grain boundary can be estimated using the following equations.

First, the total wetted area of the droplet, A_{total}, is given by

$$A_{total} = \pi(\frac{d}{2})^2 . \qquad \text{(S9-1)}$$

Then, the area taken up by the grain boundary, A_{GB}, can be estimated as a rectangle with length given by the diameter of the droplet, and width aligning with the physical width of the electrochemically active region of the grain boundary. This yields:

$$A_{GB} = dw . \qquad \text{(S9-2)}$$

These parameters and areas are shown graphically in Figure S9-1.

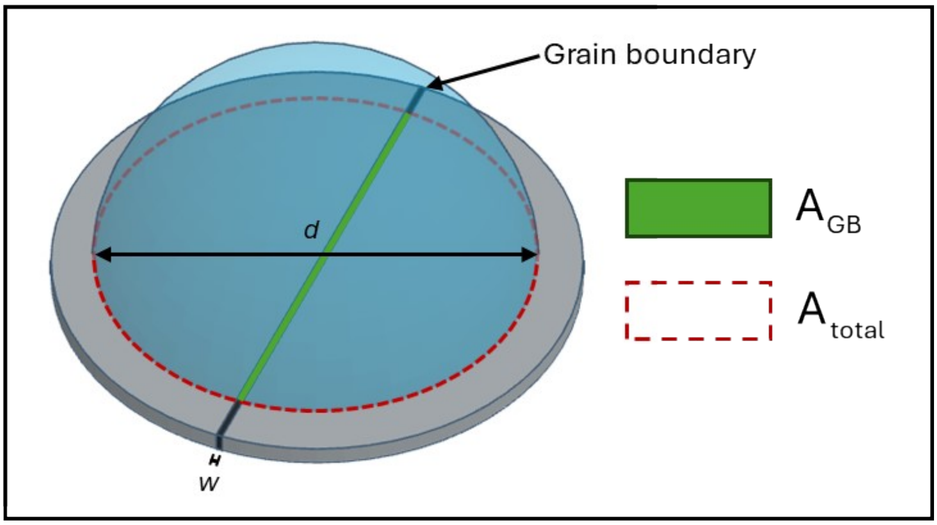

Figure S9-1: Graphical depiction of defined parameters d, w, A_{total} and A_{GB} in an SECCM droplet cell. The droplet is shown sitting on a working electrode surface, with a grain boundary line present in the centre of the contact area of the droplet.

The percentage of the droplet area taken up by the grain boundary, $\%_{GB}$, is then found by combining Equations S9-1 and S9-2 with

$$\%_{GB} = \frac{A_{GB}}{A_{total}} \times 100$$

$$\%_{GB} = \frac{4w}{\pi d} \times 100 . \qquad \text{(S9-3)}$$

It should be noted that these estimations assume a grain boundary width much smaller than the droplet diameter, and that the grain boundary is straight feature with uniform thickness that passes through the

centre of the droplet. Whilst these assumptions certainly do not apply to all cases, they are adequate for basic estimations.

In this study, with droplet diameters on the order of 250 nm, and grain boundary widths estimated to be approximately 2 nm, the percentage of the droplet areas taken up by a grain boundary on a successful boundary landing would then follow

$$\frac{4w}{\pi d} \times 100 = \frac{4(2 \times 10^{-9})}{\pi(250 \times 10^{-9})} \times 100 = 1\% .$$

As such, the fractional area of a grain boundary in an SECCM droplet cell applicable to this study is quoted as 1% in the main text.

Section S10 – Estimation of parameters for realistic detectability of grain boundaries by SECCM

To make a meaningful detection of an 'active' grain boundary, the current obtained on the working electrode area within an SECCM droplet cell at a given potential must be higher than the surrounding areas without grain boundaries present. For the sake of convenience, a 10% increase in current density on grain boundary landings is considered to be a reasonable limit of detection by which one may be confident in the fact that they have found a meaningful increase in activity. To determine the requirements for a grain boundary to yield such an increase in current response, the following calculations are performed.

For a given potential that leads to HER processes, it can be assumed that the bulk surface (containing no grain boundaries) and the active region of the grain boundary each have unique and consistent current densities. These will be referred to as j_{bulk} and j_{GB} respectively. For a grain boundary that is more active than the surrounding bulk for the HER, we can define a parameter γ (referred to in the main text) to be the ratio of these current densities,

$$\gamma = \frac{j_{GB}}{j_{bulk}}, \qquad \text{(S10-1)}$$

which will yield a value larger than unity.

Using this parameter γ, the current obtained from an SECCM landing can be found for cases with a grain boundary present or absent from the analysed surface. Starting with the absence of a grain boundary, the obtained current is

$$i_{no\ GB} = j_{bulk} \times A_{total}.$$

And, with the total droplet area, A_{total}, given by Equation S9-1, this becomes

$$i_{no\ GB} = j_{bulk} \times \pi(\frac{d}{2})^2 . \qquad \text{(S10-2)}$$

The current obtained from a location containing a grain boundary involves slightly more involved calculation, as it includes contributions from both the grain boundary itself and the surrounding bulk surface. In this case, the total droplet landing area, A_{total}, consists of the grain boundary line, A_{GB}, and the remaining area of the bulk, A_{bulk}. A_{total} and A_{GB} are defined in Equations S10-1 and S10-2, whilst A_{bulk} can be found by subtracting the latter from the former. With the two area components defined, the overall current is found by the sum of these areas, each multiplied by their respective current densities. This is found by

$$i_{GB\ present} = j_{bulk} \times A_{bulk} + j_{GB} \times A_{GB}$$

$$i_{GB\ present} = j_{bulk} \times (A_{total} - A_{GB}) + j_{GB} \times A_{GB}$$

$$i_{GB\ present} = j_{bulk} \times (\pi(\frac{d}{2})^2 - dw) + j_{GB} \times dw .$$

And, if j_{GB} is defined in terms of j_{bulk} using γ, this becomes

$$i_{GB\ present} = j_{bulk} \times (\pi\left(\frac{d}{2}\right)^2 - dw) + (\gamma \times j_{bulk}) \times dw$$

$$i_{GB\ present} = j_{bulk} \times d(\frac{\pi d}{4} + w(\gamma - 1)) . \qquad \text{(S10-3)}$$

Now, since it has already been established that the current on a grain boundary-including landing must be 10% higher than the current on a landing with no grain boundary in order for the difference in activity to be detectable, this necessitates

$$\frac{i_{GB\ present}}{i_{no\ GB}} \geq 1.1 \quad . \tag{S10-4}$$

Substituting Equations S10-2 and S10-3 into Equation S10-4 gives

$$\frac{j_{bulk} \times d(\frac{\pi d}{4} + w(\gamma - 1))}{j_{bulk} \times \pi(\frac{d}{2})^2} \geq 1.1$$

$$1 + \frac{4w(\gamma - 1)}{\pi d} \geq 1.1 \quad , \tag{S10-5}$$

which defines the necessary condition for grain boundary activity to be detectable by SECCM, based on the parameters w, d, and γ. This relationship is shown graphically in Figure 7 of the main text.

Rearranging Equation S10-5 to make γ the subject of the equation yields

$$\gamma \geq 1 + \frac{\pi d}{40w} . \tag{S10-6}$$

For any experiment where d and w are known, Equation S10-6 then estimates the lowest possible value of γ such that grain boundary activity can be detected. For this study, d and w are estimated to be 250 nm and 2 nm respectively, so

$$\gamma \geq 1 + \frac{\pi(250 \times 10^{-9})}{40(2 \times 10^{-9})}$$

$$\gamma \geq 10.8 \ .$$

Hence, under the parameters of the analysis undertaken in this study, the current density on the active region of a grain boundary needs to be at least ten times higher than the current density on the surrounding surface if grain boundary activity were to be detected. This approximate value is quoted in the main text. Since grain boundary activity was not observed, it can be concluded that grain boundary activity for the acidic HER on platinum either does not exist, or it falls below this value.

Section S11 – Use of a leakless Ag|AgCl reference electrode as a counter-reference electrode in a two-electrode system

In scans provided in Figures 5 and 6 of the main text (alongside all figures given in Sections S1 and S6), the SECCM setup utilised a leakless miniature Ag|AgCl reference electrode (eDAQ, Australia) as a counter-reference electrode. In this case, the resulting two-electrode system forced the Ag|AgCl reference to conduct current equal in magnitude to that on the working electrode during measurement. Of course, forcing a reference electrode to conduct current directly in this way can generally lead to some degree of offset in its potential, since no electrode can exhibit ideally non-polarisable behaviour.

To demonstrate the validity of the reference potential defined by this electrode when conducting current of the magnitudes encountered in SECCM, Figure S11-1 is provided. Demonstrated within is the resulting potential offset of the electrode (from its nominal potential) caused by actively driving constant current through the reference electrode directly for 60 seconds (after which time, the potential of the electrode was observed to reach equilibrium at its reported 'offset' value). It is observed that potential offset of this kind is negligible when the magnitude of applied current is less than 10^{-8} A. Since measured current magnitudes were never more than 10^{-8} A in any provided SECCM scan, it is assumed that the reference potentials referred to were not subject to meaningful drift.

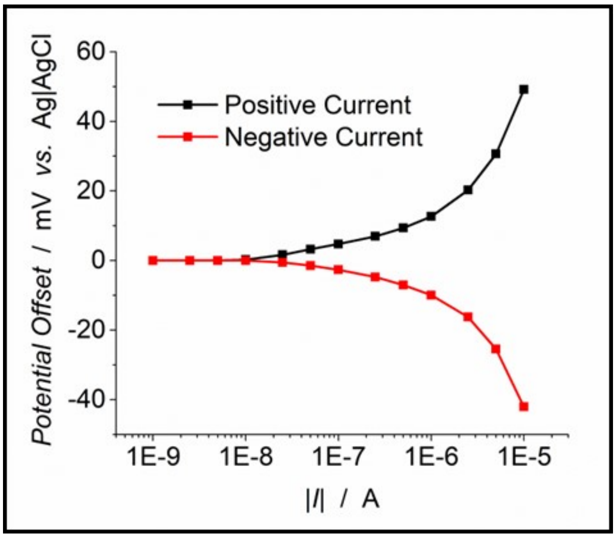

Figure S11-1: Potential offset of the utilised leakless Ag|AgCl electrode when forced to draw constant current directly. It is observed that the reference potential is very stable when conducting current lower in magnitude than the 10^{-8} A range.

References:

(1) Sutton, A. P.; Banks, E. P.; Warwick, A. R. The Five-Dimensional Parameter Space of Grain Boundaries. *Proc. R. Soc. Math. Phys. Eng. Sci.* **2015**, *471* (2181), 20150442. https://doi.org/10.1098/rspa.2015.0442.

(2) Hu, C.; Zuo, Y.; Chen, C.; Ping Ong, S.; Luo, J. Genetic Algorithm-Guided Deep Learning of Grain Boundary Diagrams: Addressing the Challenge of Five Degrees of Freedom. *Mater. Today* **2020**, *38*, 49–57. https://doi.org/10.1016/j.mattod.2020.03.004.

(3) Chesser, I.; Francis, T.; De Graef, M.; Holm, E. A. Learning the Grain Boundary Manifold: Tools for Visualizing and Fitting Grain Boundary Properties. *Acta Mater.* **2020**, *195*, 209–218. https://doi.org/10.1016/j.actamat.2020.05.024.

(4) Saylor, D. M.; Morawiec, A.; Rohrer, G. S. Distribution and Energies of Grain Boundaries in Magnesia as a Function of Five Degrees of Freedom. *J. Am. Ceram. Soc.* **2002**, *85* (12), 3081–3083. https://doi.org/10.1111/j.1151-2916.2002.tb00583.x.

(5) Bulatov, V. V.; Reed, B. W.; Kumar, M. Grain Boundary Energy Function for Fcc Metals. *Acta Mater.* **2014**, *65*, 161–175. https://doi.org/10.1016/j.actamat.2013.10.057.

(6) Ye, W.; Zheng, H.; Chen, C.; Ong, S. P. A Universal Machine Learning Model for Elemental Grain Boundary Energies. *Scr. Mater.* **2022**, *218*, 114803. https://doi.org/10.1016/j.scriptamat.2022.114803.

(7) Britton, T. B.; Jiang, J.; Guo, Y.; Vilalta-Clemente, A.; Wallis, D.; Hansen, L. N.; Winkelmann, A.; Wilkinson, A. J. Tutorial: Crystal Orientations and EBSD — Or Which Way Is Up? *Mater. Charact.* **2016**, *117*, 113–126. https://doi.org/10.1016/j.matchar.2016.04.008.

(8) Baik, S.-I.; Olszta, M. J.; Bruemmer, S. M.; Seidman, D. N. Grain-Boundary Structure and Segregation Behavior in a Nickel-Base Stainless Alloy. *Scr. Mater.* **2012**, *66* (10), 809–812. https://doi.org/10.1016/j.scriptamat.2012.02.014.

(9) Randle, V. Overview No. 127The Role of the Grain Boundary Plane in Cubic Polycrystals. *Acta Mater.* **1998**, *46* (5), 1459–1480. https://doi.org/10.1016/S1359-6454(97)00338-8.

(10) Wang, Y.; Li, M.; Ren, H. Interfacial Structure and Energy Determine the Heterogeneity in the Electrochemical Metal Dissolution Activity at Grain Boundary. *Chem. Mater.* **2023**, *35* (11), 4243–4249. https://doi.org/10.1021/acs.chemmater.3c00220.

(11) Sheppard, S.-A.; Campbell, S. A.; Smith, J. R.; Lloyd, G. W.; Walsh, F. C.; Ralph, T. R. Electrochemical and Microscopic Characterisation of Platinum-Coated Perfluorosulfonic Acid (Nafion 117) Materials†. *Analyst* **1998**, *123* (10), 1923–1929. https://doi.org/10.1039/A803310B.
